# Regulatory (pan-)genome of an obligate intracellular pathogen in the PVC superphylum

**DOI:** 10.1038/ismej.2016.23

**Published:** 2016-03-08

**Authors:** Marie de Barsy, Antonio Frandi, Gaël Panis, Laurence Théraulaz, Trestan Pillonel, Gilbert Greub, Patrick H Viollier

**Affiliations:** 1 Institute of Microbiology, University Hospital Center, University of Lausanne, Lausanne, Switzerland; 2 Department of Microbiology and Molecular Medicine, Institute of Genetics and Genomics in Geneva (iGE3), Faculty of Medicine, University of Geneva, Geneva, Switzerland

## Abstract

Like other obligate intracellular bacteria, the *Chlamydiae* feature a compact regulatory genome that remains uncharted owing to poor genetic tractability. Exploiting the reduced number of transcription factors (TFs) encoded in the chlamydial (pan-)genome as a model for TF control supporting the intracellular lifestyle, we determined the conserved landscape of TF specificities by ChIP-Seq (chromatin immunoprecipitation-sequencing) in the chlamydial pathogen *Waddlia chondrophila*. Among 10 conserved TFs, Euo emerged as a master TF targeting >100 promoters through conserved residues in a DNA excisionase-like winged helix-turn-helix-like (wHTH) fold. Minimal target (Euo) boxes were found in conserved developmentally-regulated genes governing vertical genome transmission (cytokinesis and DNA replication) and genome plasticity (transposases). Our ChIP-Seq analysis with intracellular bacteria not only reveals that global TF regulation is maintained in the reduced regulatory genomes of *Chlamydiae*, but also predicts that master TFs interpret genomic information in the obligate intracellular α-proteobacteria, including the rickettsiae, from which modern day mitochondria evolved.

## Introduction

Regulation at the level of transcription initiation represents the most commonly used strategy for cellular reprogramming and is generally orchestrated by one or several master (global) transcriptional factors (TFs) that directly coordinate the activity of hundreds of genes by binding to their promoters (Laub *et al.*, 2007; Cole and Young, 2008; Losick and Desplan 2008; Whyte *et al.*, 2013; Wolanski *et al.*, 2014; Panis *et al.*, 2015). Despite the apparent simplicity of bacteria and their compact genome size compared with their eukaryotic counterparts, free-living bacteria typically encode hundreds of TFs in their genomes. Obligate intracellular bacteria such as the *Chlamydiae* encode far fewer TFs in their reduced genomes, making them ideal models for determining the ‘regulatory pan-genome' during intracellular growth. We defined here the regulatory pan-genome as the genomic regulatory sites that are targeted by TFs common to all members of the phylum *Chlamydiae*. In other bacteria, the regulatory program implements stochastic and/or deterministic cell fate and transcriptional switches that promote productive acute or chronic infections of host cells (Arnoldini *et al.*, 2014; Deghelt *et al.*, 2014; Diard *et al.*, 2014; Manina *et al.*, 2015; Panis *et al.*, 2015). However, defining the landscape of TF specificities within the host is challenging in the case of facultative bacterial pathogens that also grow outside cells. Obligate intracellular bacteria may thus present an optimal system for this goal.

*Chlamydiaceae* family, the most described in the *Chlamydiae* phylum, includes members able to infect a wide host range (Coulon *et al.*, 2012; Kebbi-Beghdadi *et al.*, 2011, 2015) and that are well-characterized human and animal pathogens with important zoonotic implications (Wheelhouse and Longbottom, 2012). Although the estimated incidence of sexually-transmitted infections by *Chlamydia trachomatis* is >100 million per year (Baud *et al.*, 2008), other members of the *Chlamydiaceae* family, such as *Chlamydia pneumoniae*, are also pathogenic towards humans, being implicated in lung infections (Grayston, 2000; Baud *et al.*, 2008; Senn *et al.*, 2011; Taylor *et al.*, 2014). In addition to the *Chlamydiaceae*, several new *Chlamydia*-related bacteria, including *Waddlia chondrophila*, were recently discovered in diverse environments (Dilbeck *et al.*, 1990; Fritsche *et al.*, 1993; Kahane *et al.*, 1995; Amann *et al.*, 1997; Rurangirwa *et al.*, 1999; Collingro *et al.*, 2005; Thomas *et al.*, 2006; Lienard *et al.*, 2011). Some of these are harmless symbionts of ameobae, while others are implicated as emerging pathogens for humans and animals (Greub and Raoult, 2004; Lamoth and Greub, 2010). The ecological niche as well as the potential reservoirs and vectors of these bacteria are not well described yet, despite some hints suggesting the role of ticks as potential vectors (Croxatto *et al.*, 2014; Pilloux *et al.*, 2015), as well as reports supporting a role of free-living amoebae as widespread reservoir of these *Chlamydia*-related bacteria in water environment (Thomas *et al.*, 2006; Corsaro *et al.*, 2009). Moreover, the complexity and diversity of the *Chlamydiae* phylum is largely underestimated as suggested recently by metagenomic and phylogenetic analyses revealing 181 putative families present mainly in marine environments (Lagkouvardos *et al.*, 2014). Members of the *Chlamydiae* phylum are related to free-living aquatic bacteria that belong to the *Verrucomicrobia* and *Planctomycetes* (PVC superphylum) (Jackson and Weeks, 2008; Satinsky *et al.*, 2015) and are mostly (currently) genetically intractable. Interestingly, at least one member of the *Verrucomicrobia* has recently been identified as an important constituent of the human microbiota controlling obesity, brown fat tissue and cold tolerance (Chevalier *et al.*, 2015).

**Figure 1:**
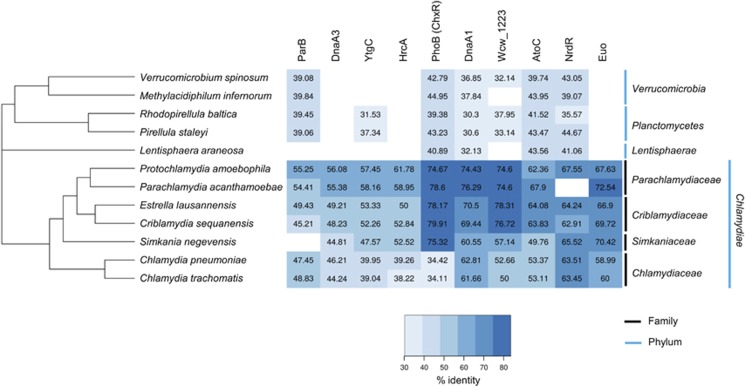
The 10 conserved TFs in *Waddlia chondrophila* and their orthologues in the PVC superphylum. Presence (blue) or absence (white) of the 10 TFs in the PVC superphylum. The % of identity, compared with *W. chondrophila*, is indicated in each square. The proteins are indicated on the top, organisms on the left and the family (black) and the phylum (blue) on the right. Organisms are ordered on the basis of the phylogenetic tree performed with the maximum likelihood method using the 158 core genes. The topology of the *Chlamydiae* phylum derived from the concatenation of the 158 core genes is similar to what is already known (Pillonel *et al*, 2015). Euo, HrcA and DnaA3 are only present in the *Chlamydiae*. PhoB, AtoC and NrdR are conserved in the PVC superphylum. ParB is not present in *Simkania negevensis* and NrdR is not present in *Parachlamydia acanthamoebae*.

The members of the *Verrucomicrobia*, *Planctomycetes* and of most chlamydial families (for example, *W. chondrophila)* have a larger genome and therefore presumably an expanded metabolic capacity (Greub *et al.*, 2009; Bertelli *et al.*, 2010) compared with the 1–1.2 Mbp genome of *C. trachomatis* and *C. pneumoniae* (Stephens *et al.*, 1998; Shirai *et al.*, 2000; Collingro *et al.*, 2011). Nevertheless, all chlamydial genomes encode a limited number of TFs (Stephens *et al.*, 1998; Shirai *et al.*, 2000; Collingro *et al.*, 2011; Siegl and Horn, 2012) (see below and Figure 1) to control the interactions with different eukaryotic hosts and an infectious cycle involving two morphotypes (Rockey and Matsumoto, 2000; Siegl and Horn, 2012; Tan, 2012). The cycle can be divided into three stages. In the first, an infectious non-dividing elementary body (EB) enters the host cells and differentiates into the non-infectious and replicative reticulate body (RB). The RB features a de-condensed genome and expresses cytokinetic proteins, to permit rapid proliferation and division of RBs within a vacuole (called the inclusion) during the mid-stage. In the late stage, the RBs differentiate back into EBs and are released by extrusion or cell lysis (Moulder, 1991). Changes in the chlamydial transcriptome are thought to underlie these developmental stages, reflected by three corresponding temporal transcript classes (early, mid and late) (Shaw *et al.*, 2000; Belland *et al.*, 2003; Nicholson *et al.*, 2003). Thus, by analogy to other developmental systems, we speculate that one or several conserved master TFs target promoters of developmentally-regulated genes.

At least 10 conserved TFs (Figure 1, note that TFs are defined here as proteins that are not predicted to be structural components of RNA polymerase holoenzyme, RNAP, or regulators of RNAP enzymatic activity) are predicted within the *Chlamydiae* (Greub *et al.*, 2009; Bertelli *et al.*, 2010). As the chlamydial TF regulatory network remains experimentally untested, we unmasked the regulatory pan-genome defined by these 10 TFs by chromatin-immunoprecipitation followed by deep-sequencing (ChIP-Seq) hoping to learn whether these 10 conserved TFs and/or a possible master TF might control the developmental and obligate intracellular proliferation cycle of chlamydia. The immunochemistry of ChIP-Seq has the advantage of minimizing the contaminating host nucleic acids compared with chlamydial transcriptome studies (Albrecht *et al.*, 2010, 2011) and, within one stroke, records the TF landscape of an obligate intracellular bacterium within its host cell.

## Materials and methods

### Cell culture and bacterial strains

Vero cells (ATCC CCL-81) were cultivated in Dulbecco's modified minimal essential medium (DMEM; GE Healthcare, Pasching, Austria) supplemented with 10% fetal bovine serum (GE Healthcare), at 37 °C in the presence of 5% CO_2_.

*Waddlia chondrophila* strain ATCC VR-1470 was co-cultivated at 32 °C with *Acanthamoeba castellani* strain ATCC 30010 in 75 cm^2^ flask containing 30 ml of peptone-yeast-extract-glucose broth. After 7 days of culture, the suspension was filtered on 5 μm filter to eliminate trophozoites and cysts and to isolate bacteria in the filtrate. The filtrate was then diluted at the appropriate dilution in DMEM to proceed to infection of Vero cells.

### Infection procedure

One day before the infection, 5 × 10^5^ Vero cells were seeded per well in 24-well plates. A 1/2000 dilution of *W. chondrophila* was used to infect Vero cells. This corresponded to a multiplicity of infection 2–3, as estimated by qPCR. This infectious dose led to 50% of infected cells with 2–3 bacteria, as determined by confocal microscopy. The infected Vero cell suspension was centrifuged at 1790 *g* for 10 min at room temperature and then incubated for 15 min at 37 °C with 5% CO_2_. To remove non-internalized bacteria to obtain a synchronous infection, cells were washed with the DMEM. Then, infected cells were incubated for different time periods.

### Quantitative PCR

The number of bacteria at different time post-infection was determined using real-time quantitative PCR (qRT-PCR). Infected cells were recovered after cell scraping. Genomic DNA extraction was performed on 50 μl of cell suspension using the WizardSV Genomic DNA purification kit (Promega, Madison, WI, USA) and eluted in 100 μl. The quantitative PCR was performed as described previously using iTaq supermix with ROX (Bio-Rad, Reinach, Switzerland) (Goy *et al.*, 2009). The reaction mixture contained 10 μl of iTaq supermix, 200 nm of primers WadF4 and WadR4, 100 nm of the probe WadS2 and 5 μl of DNA. The qPCRs were performed on the Step-One system (Applied Biosystems, Zug, Switzerland) using the following cycling conditions: 3 min at 95 °C, 40 cycles of 15 s at 95 °C and 1 min at 60 °C.

### Crosslinking experiments

Five μm of His_6_-Euo was incubated, during 15 min at 25 °C, in the presence or not of 1 mm of bis(maleimido)hexane (Pierce, Rockford, IL, USA) a crosslinking agent. When indicated, 80 ng of PCR amplified DNA fragment was added to the reaction mix. Four μl of 6 × denaturing Laemmli loading dye was added to each sample. The samples were then separated by SDS-PAGE on a 12% polyacrylamide gel (Bio-Rad) and transferred onto nitrocellulose. The His_6_-Euo was detected by immunodetection using mouse monoclonal anti-His_6_ (Sigma-Aldrich, St Louis, MO, USA) (see Immunoblotting section).

### Data access, supplementary data sets and supplementary information

All ChIP-Seq data files have been deposited at the GEO database under accession number GSE68059. Supplementary tables, figures and additional experimental procedures are available for download from the *ISME Journal* website.

## Results

### Conservation of TFs within the PVC

To investigate the conservation of chlamydial TFs, we first identified homologues of 10 putative *W. chondrophila* TFs in all known chlamydial genera and representatives of two sister clades of the *Chlamydiae* phylum, the *Planctomycetes* and *Verrucomicrobia*. We then conducted pairwise sequence comparisons between the 12 members of the PVC superphylum. This revealed that several of these 10 TFs are conserved within the entire PVC superphylum (PhoB, AtoC, DnaA1, NrdR and to a lesser extent ParB), while others are not (DnaA3, HrcA and Euo). Euo may have a key role in *Chlamydiae* as it is the only conserved TF unique to the *Chlamydiae* and shows a remarkable degree of sequence conservation (>70% identity in some cases, Figure 1). Wcw_1223 is present within the PVC and in several representatives of the *Bacteroidetes* phylum, albeit with a significantly lower (ca. 35%) sequence identity level. By contrast, the paralogues of the bifunctional replication initiator/TF DnaA and orthologues of the heat-sensitive repressor HrcA are also found outside the PVC lineage. The phosphate response regulator PhoB is also found in other lineages, but it is unusual because it features a high degree of conservation (near 80% identity) across many chlamydial families (especially those that replicate in amoebae), except for the *Chlamydiaceae* family (Figure 1). As the sequence identity between *W. chondrophila* PhoB and the orthologues encoded in *Planctomycetes*, *Verrucomicrobia*, *Lentisphaerae* and the other *Chlamydiaceae* genomes is considerably lower, we hypothesize that this reflects a functional specialization of PhoB within the chlamydia to control a distinct regulon. This notion and that Euo is a master chlamydial TF gained further supported by the ChIP-Seq experiments described below.

### Developmental regulation of TFs in *W. chondrophila*

**Figure 2:**
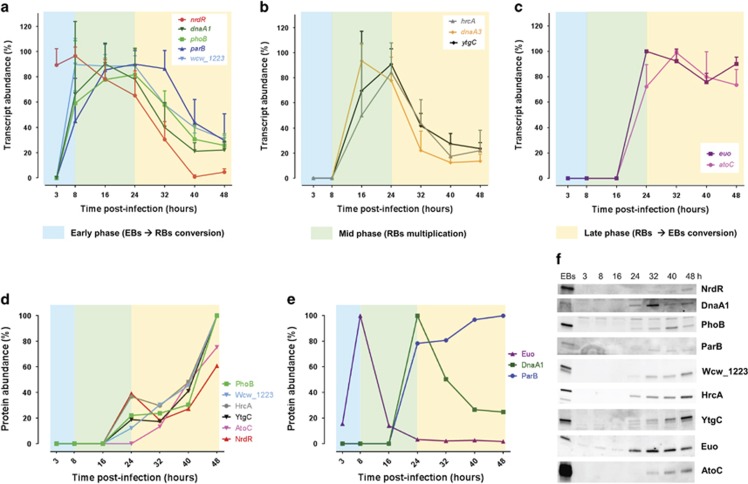
Temporal expression of the 10 TFs during the developmental cycle of *W. chondrophila*. Samples (equal volume) of infected Vero cells were harvested at different times p.i. and analysed by qRT-PCR (**a**–**c**) or by immunoblotting using specific polyclonal antibodies to the TFs (**d**–**f**). The transcript abundance (%) during the developmental cycle (early (blue shading), mid (green) and late (yellow)) was determined for each TF (**a**–**c**). The data are shown as mean values±standard deviation of three independent experiments. Most of the genes show a mid-phase transcript profile (**a**, **b**) except for *euo* and *atoC*, which exhibit a late-phase transcript profile (**c**). The TF abundance (%, **d** and **e**) was quantified by normalization of the signal detected on the blot (**f**) according to the number of bacteria/well defined by qPCR. Most of the TFs accumulated steadily along the progression of the developmental cycle (**d**). Euo and DnaA1 exhibited a peak in abundance at 8 h and 24 h, respectively (**e**). Note that we could not unambiguously detect DnaA3 by immunoblotting and thus omitted it from this analysis.

To determine whether these conserved TFs are temporally regulated during the developmental cycle of *W. chondrophila*, we measured their transcript levels by qRT-PCR at various time points during intracellular growth, that is, post infection (p.i.) of Vero cells with *W. chondrophila*. We observed a peak in the steady-state levels of the *dnaA1*, *phoB*, *parB*, *wcw_1223*, *hrcA*, *dnaA3* and *ytgC* transcripts in mid-phase (16–24 h p.i., Figures 2a and b). By contrast, the *nrdR* transcript levels are high at 3 h and 8 h p.i. (Figure 2a), followed by a progressive drop until 40 h p.i. The two remaining transcripts, *euo* and *atoC*, were only detected from 24 h p.i. onwards and remained high henceforth (Figure 2c), suggesting that these TFs are important late in the current developmental cycle or may be needed for an early event in the ensuing cycle.

To determine the relative TF protein abundance during the developmental cycle, we conducted immunoblotting (see Materials and methods) using polyclonal antibodies to each of these TFs (Figures 2d–f). We observed a relative increase in abundance of NrdR, AtoC, YtgC, HrcA, Wcw_1223 and PhoB along the developmental cycle (Figures 2d and f). By contrast, ParB levels surged at 24 h p.i. and then plateaued (Figures 2e and f), Euo and DnaA1 peaking at 8 h and 24 h p.i., respectively. The abundance of the Euo protein at an early time point is surprising considering that its transcript abundance is highest from 24 h onwards. One possibility is that the *euo* transcript is synthesized during the conversion of RBs into EBs (late phase) and stored in EBs, thus allowing immediate translation during the early stage of the infection in the next cycle. A similar peak in *euo* transcripts has been observed for *C. psittaci euo* at 15 h p.i. (Zhang *et al.*, 1998), suggesting that the developmental control of *euo* in different chlamydial families is conserved. It is also possible that the translation and/or stability of Euo is differentially regulated.

### Landscape of TF specificities in *W. chondrophila*

**Figure 3:**
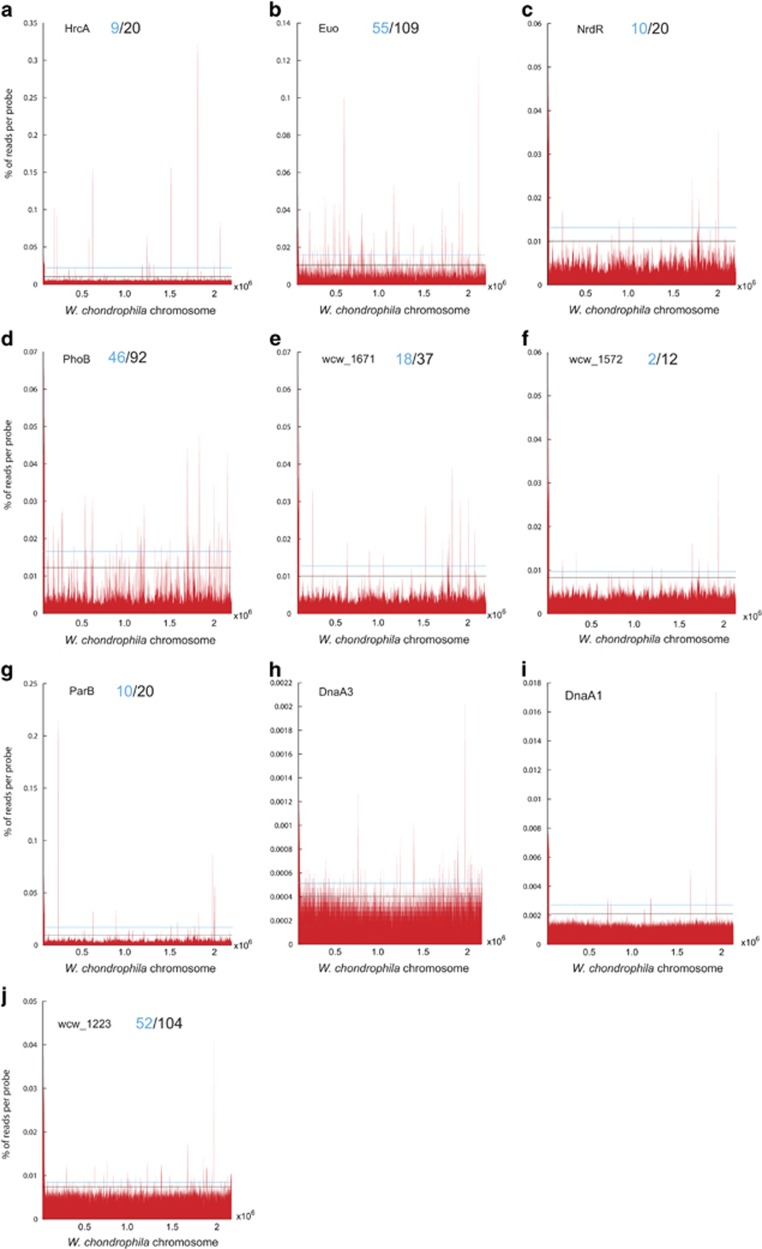
Occupancy of the 10 conserved TFs on the *W. chondrophila* genome. (**a**–**j**) ChIP-Seq profiles of the 10 TFs. Black line on the graphs depicts the cutoff used to identify the total predicted targets of each TF, while the blue line denotes the median score used to select the predicted high confidence targets (blue) from the total predicted target sites (black). The coordinates below the graph (x axis) indicate the nucleotide (nt) position along the *W. chondrophila* genome and the y axis shows the relative abundance of the corresponding nt position in the precipitated sample. Owing to the poor quality of the DnaA1 and DnaA3 precipitates we elected not to predict targets.

To chart the landscape of TF specificities, we conducted ChIP-Seq experiments in *W. chondrophila* with the antibodies to the TFs (Supplementary Figure 2A–C, Supplementary Table 1). Using our peak-finding strategy (see Supplementary Information), we observed a range of total TF target sites from >100 to <10 in some cases at different levels of enrichment (that is, enrichment of 0.5–1 times above the 2 s.d. cutoff, Figures 3a–f). We defined a subset of high confidence targets within in each set using the median value of relative abundance as a reference point compared with the standard 2 s.d. cutoff value (Figures 3a–f), with the predicted targets for Euo, for example, lying primarily in putative promoters or intergenic regions (Supplementary Figure 2D–F). Our analysis predicted 9/18 (HrcA), 55/109 (Euo), 46/92 (PhoB) high confidence/total sites within promoter regions (400 bp upstream to 100 bp downstream of the start codon of a predicted coding sequence, Figures 3a, b and d; Supplementary Table 2). The high confidence target lists for HrcA contains genes such as the *groES* and *groEL* chaperone genes, which are targets of the *C. trachomatis* HrcA orthologue *in vivo* (Chen *et al.*, 2011; Rosario and Tan, 2012; Domman and Horn, 2015; Hanson and Tan, 2015). By contrast, no putative target sites are known for PhoB in other *Chlamydiae*. Fewer conserved PhoB targets were found for the *Chlamydiaceae* family than for other families (Supplementary Table 1). Importantly, we successfully derived distinct consensus motifs by Multiple Em for Motif Elicitation (MEME)-based analyses (see Supplementary Information, Supplementary Figure 2A–C) from the target lists of these three TFs (Euo, HrcA and PhoB) and validated them as described below.

### Validation of HrcA and PhoB target sites

**Figure 4:**
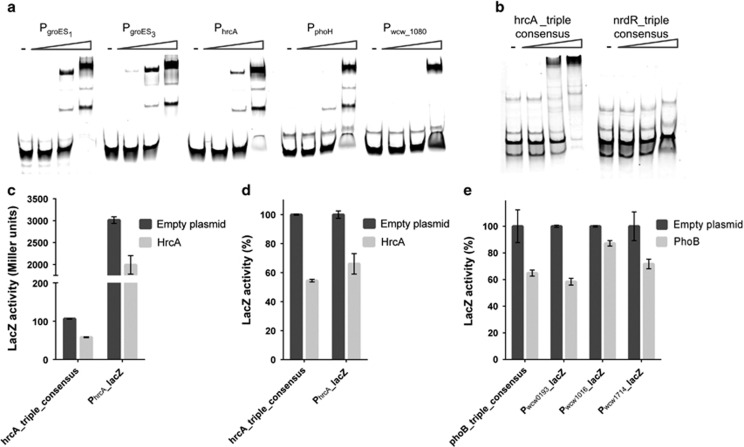
ChIP-Seq validation for HrcA and PhoB. (**a**) Binding of His_6_-HrcA on promoters identified by ChIP-Seq (P_*groES1*_, P_*groES3*_, P_*hrcA*_, P_*phoH*_ and P_*wcw_1080*_). The promoters were amplified by PCR using specific primers coupled to Cy5. DNA fragments were incubated in the absence (−) or in presence of an increasing concentration of His_6_-HrcA (50, 250 and 1250 nm) and analysed by EMSA. (**b**) EMSA analysis showing the binding of His_6_-HrcA to a synthetic fragment harbouring the HrcA-triple consensus. As a negative control, we used an analogous triple-repeat fragment harbouring the predicted NrdR consensus motif. (**c**, **d**) *In vivo* binding of HrcA on the triple consensus and on its own promoter (P_*hrcA*_) using *lacZ* reporter gene in *E. coli*. HrcA was expressed from an arabinose-inducible promoter on pBAD22 (Guzman *et al.,* 1995). As a negative control, the expression empty vector was used. Data are means±standard deviation of three biological triplicates. Panel (**c**) absolute values, panels (**d**) and (**e**) normalized value according to the empty plasmid (set as 100%). When HrcA was expressed, a decrease of 40% of the LacZ activity was observed, suggesting that HrcA is able to bind and to repress the expression of *lacZ*. (**e**) *In vivo* binding of PhoB (expressed from pBAD22) on three promoters (P_*wcw_0193*_, P_*wcw_1016*_ and P_*wcw_1714*_) identified by ChIP-Seq and on the PhoB-triple consensus using *lacZ* reporter gene in *E. coli.* A decrease of the LacZ activity was observed for the four *lacZ* promoter-probe plasmids.

Five out of the nine predicted high confidence *in vivo* targets for HrcA (Figure 3a) were selected for testing by *in vitro* *e*lectrophoretic *m*obility *s*hift *a*ssay (EMSA) with a purified recombinant His_6_-tagged version of *W. chondrophila* HrcA (His_6_-HrcA) (see Materials and methods) and fluorescently-labelled target promoter fragments (P_*hrcA*_, P_*groES1*_, P_*groES3*_, P_*phoH*_ and P_*wcw_1080*_) as probes. All five probes were band-shifted by His_6_-HrcA (Figure 4a). Since the consensus motif for HrcA deduced above (Supplementary Figure 2C; Supplementary Table 2, 5′TAGCA-(N)_15_-TGCTAA-3′) matches the CIRCE element-containing inverted repeat that is bound by HrcA in other bacteria and that predicted for *C. trachomatis* (Hecker *et al.*, 1996; Narberhaus, 1999; Minder *et al.*, 2000; Wilson and Tan, 2004; Hanson and Tan, 2015), we tested whether His_6_-HrcA also band-shifts a synthetic fragment harbouring a triple repeat of our HrcA consensus motif (Figure 4b). This was the case, but not for an analogous synthetic fragment harbouring a triple repeat of the unrelated NrdR consensus motif (see Supplementary Information, Figure 4b).

Having validated the HrcA target motif *in vitro*, we next tested if HrcA binds the consensus motif *in vivo* in a surrogate host. To this end, we used *an E. coli* β-galactosidase (LacZ)-based transcription interference assay in which a synthetic promoter containing the triple repeat of the HrcA consensus motif directs the expression of a promoterless *lacZ* on a low-copy plasmid in *E. coli*. We used this plasmid to test whether *W. chondrophila* HrcA expressed heterologously in *E. coli* can downregulate promoter activity (measured as LacZ activity). We observed that LacZ activity dropped by 40% when HrcA was expressed compared with *E. coli* cells harbouring the empty vector (Figures 4c and d). Since ChIP-Seq suggested autoregulation of HrcA, we conducted a similar LacZ-based interference assay in *E. coli* with native *W. chondrophila* P_*hrcA*_ promoter using the P_*hrcA*_-*lacZ* reporter plasmid (Figures 4c and d). A commensurate decrease of ±35% of the LacZ activity compared with the empty vector was also seen for P_*hrcA*_, establishing that HrcA also binds this site and consensus *in vivo*.

Next, we used a BBH (bidirectional best blast hit) approach to compile a list of orthologous target genes of HrcA encoded in 13 *Chlamydiales* (Supplementary Figure 3A). This list clustered into two main groups. The first cluster contains genes conserved across the *Chlamydiales* order (>40% identity) and includes genes targeted by *C. trachomatis* HrcA *in vitro* for example *groES1(ct111/wcw_1342)*, *groEL1(ct110/wcw_1343)*, *groES3(wcw_1848)*, *groEL3(wcw_1849)* and *hrcA(ct394/wcw_1636)* (Tan *et al.*, 1996; Wilson and Tan, 2004; Hanson and Tan, 2015). The second larger cluster harbours targets mostly restricted to *W. chondrophila*, including 12 genes that seem to define the accessory (specific) *W. chondrophila* HrcA target regulon (see Discussion).

Next, we validated the predicted target sites of PhoB (Supplementary Table 3). The deduced 21-bp consensus motif computed by MEME (5′-(T/A)NTN**AA(G/A)AAA**NTGN(T/A)AAATTT-3′, Supplementary Figure 2B; Supplementary Table 3) includes a sequence (in bold) resembling the predicted half site for the distantly related PhoB orthologue ChxR of *C. trachomatis* (Hickey *et al.*, 2011). As described above for HrcA, we used *an E. coli* transcriptional interference assay with the native target promoters (identified by ChIP-Seq, Supplementary Figure 2B) or a synthetic promoter harbouring a triple repeat of the PhoB consensus (PhoB-triple consensus) upstream of the promoterless *lacZ* gene and expressed PhoB from a second plasmid (arabinose-inducible promoter). We observed a reduction in LacZ activity by ±40% comparing the P_*wcw_0193*_ and P_*phoB-triple-consensus*_ and by ±15% and 30% for P_*wcw_1016*_ versus P_*wcw_1714*_ promoter-probe plasmids, respectively, compared with the empty vector (Figure 4e). Thus, *W. chondrophila* PhoB binds these sites *in vivo.* BBH-based conservation analysis (Supplementary Figure 3B) revealed a small group of PhoB-regulated genes highly conserved across the phylum *Chlamydiae* (for example, the *sctE*[*wcw_1612*] predicted to encode a needle chaperone involved in inclusion modification and in chlamydial pathogenesis), specifically within the *Chlamydia*-related bacteria. The other group of PhoB-regulated genes includes *W. chondrophila* orthologues present also in other members of the *Chlamydiae* phylum, but with a lower degree of conservation and of unknown function.

### Euo has properties of a master TF

**Figure 5:**
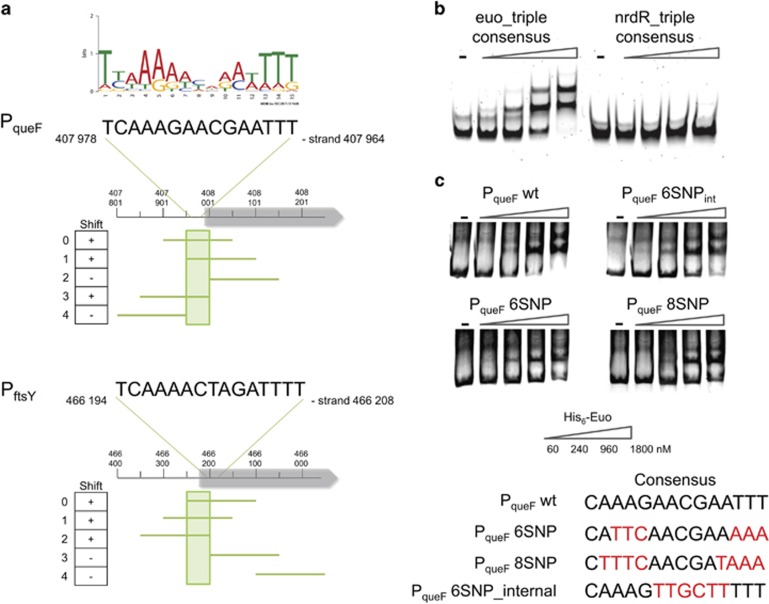
Euo consensus binding site analysis. (**a**) Identification of 50-bp region which includes the consensus and is necessary for the binding of His_6_-Euo. PCR probes shifted by 50 bp were designed for the P_*queF*_ and P_*ftsY*_ promoters and all fragments were used for *in vitro* binding assay (EMSA) with His_6_-Euo. A total of 80 ng of DNA fragments were incubated in the absence or in the presence of an increasing concentration of His_6_-Euo, protein–DNA complexes were detected using GelRed. Results are presented in the left column. + or − indicates whether or not shifted bands were observed. (**b**) EMSA analysis of His_6_-Euo binding to the Euo-triple consensus amplified with a specific primer coupled to Cy5. As a negative control, we used the triple repeat of the predicted NrdR consensus binding site (see Figure 4b). (**c**) Mutations in the consensus poorly affect the binding of His_6_-Euo tested by EMSA (GelRed detection, see Supplementary Information). His_6_-Euo concentrations used in the gel shift assay are indicated in (**c**).

Of the >100 predicted Euo target sites, 19 (of 21 tested) were band-shifted by *W. chondrophila* His_6_-Euo in EMSAs (Table 1, Supplementary Figure 4A). Moreover, probing a blotted EMSA gel with antibodies to Euo confirmed that the shifted bands were indeed His_6_-Euo-DNA nucleoprotein complexes (Supplementary Figure 4B). The Euo consensus motif predicted by MEME (5′-TTAAAAACAAATTTT-3′, Supplementary Figure 2A; Supplementary Table 4), resembles part of the proposed extended Euo binding sites (5′-AGTAG**GTAACAACCAAGTACTT**GGGTTTT-3′ and 5′-TTT**TAAAAAACAATTG**ATATAATTTTTATT-3′) deduced for *C. trachomatis* and *C. psittaci* based on targets identified *in vitro* (Zhang *et al.*, 2000; Rosario and Tan, 2012). To explore whether our *W. chondrophila* Euo consensus motif is necessary for binding by His_6_-Euo, we designed five specific EMSA probes for P_*queF*_ and P_*ftsY*_ with 50 bp shifts including (or not) the predicted Euo DNA-binding motif (Figure 5a and Supplementary Table 6). EMSAs revealed that His_6_-Euo bound only P_*queF*_, probes that include the predicted 15-bp Euo consensus motif, but not those lacking it. Interestingly, His_6_-Euo also shifted P_*ftsY*_ probes containing only a part of the Euo consensus motif (Figure 5a).

Next, we designed synthetic EMSA probes in which the Euo consensus was repeated three times (Euo-triple consensus) and showed that His_6_-Euo bound this probe, whereas no shift was observed with the NrdR-triple repeat consensus (Figure 5b). Transcriptional interference assays in *E. coli* revealed that expression of Euo decreased by 50% the LacZ activity for P_*queF*_, P_*rpoB*_, P_*rhs9*_ and P_*wcw_1705*_ and only decreased by 15–20% for P_*wcw_0666*_, P_*hrcA*_ and P_*gltT*_ (see Figure 7a). These results show that Euo binds these promoters *in vivo* in *E. coli*.

### Determinants directing Euo to its targets

Next, we used site-specific mutagenesis to identify the critical positions necessary for Euo to bind its targets. We designed mutant P_*queF*_ EMSA probes (Figure 5c, Supplementary Figure 5) carrying specific nucleotide substitutions (Figure 5c), either near the end or in the middle of the predicted Euo target motif. As His_6_-Euo still band-shifted these probes we concluded that additional determinants must exist in the context of a native *W. chondrophila* promoter (Figure 5a) to recruit His_6_-Euo *in vitro*.

**Figure 6:**
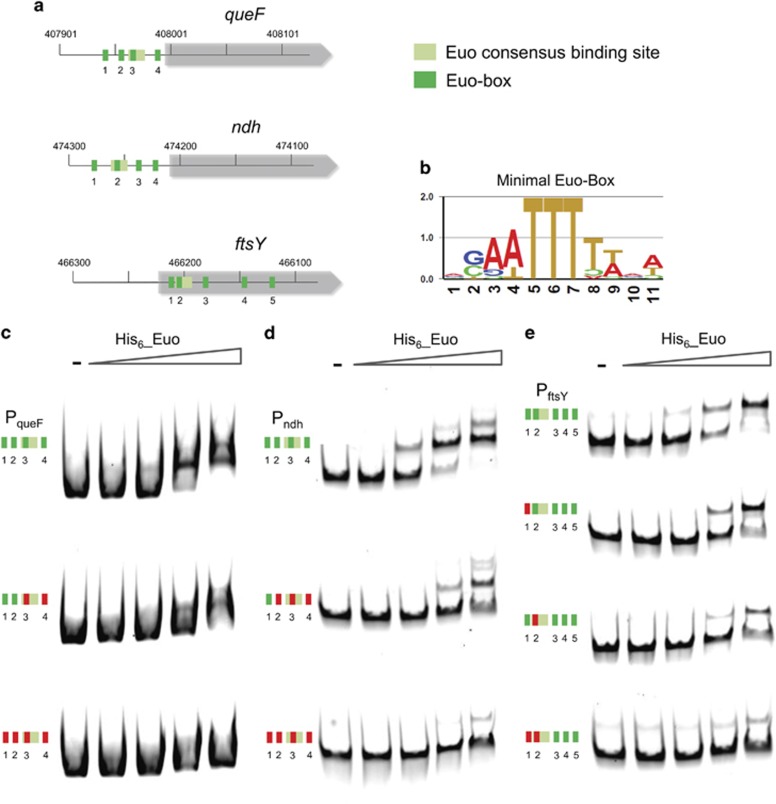
Discovery of a minimal Euo target box. (**a**) Several minimal Euo boxes (green) are present in P_*queF*_, P_f*tsY*_ and P_*ndh*_. Euo consensus binding site is also represented in light green. (**b**) Consensus based on these Euo boxes showing the conservation of the TTT. The TTT was replaced by GGG (shown in red) and the His_6_-Euo binding was tested by EMSA (c–e). The synthetic DNAs carrying the mutations were amplified using a specific primer coupled to Cy5 and used for EMSA. Mutation (red boxes) of the four Euo boxes (green) in P_*queF*_ and P_*ndh*_ completely abolished the binding of His_6_-Euo as no band-shift was observed (**c**, **d**). Mutations in the two first Euo boxes in P_*ftsY*_ completely abolished the binding of His_6_-Euo (**e**).

To identify these unknown determinants, we re-inspected the sequence of P_*queF*_, P_*ndh*_ and P_*ftsY*_ and observed the presence of a repeated, short and conserved box (3′-(A/G)(A/C)(A/T)TTT-5′, henceforth minimal Euo box). Interestingly, one minimal Euo box was always embedded in the long consensus motif predicted by MEME above (Figures 6a and b and Supplementary Figure 6). To explore the role of the minimal box in recruiting His_6_-Euo, we substituted the TTT by GGG signature one at a time and found for P_*queF*_ and P_*ndh*_ that it was necessary to mutate all four minimal Euo boxes to impede binding of His_6_-Euo (Figures 6c and d). This was not the case for P_*ftsY*_ where mutations in the first two boxes were sufficient to prevent the binding of His_6_-Euo to its target sites (Figure 6e). Thus, minimal boxes direct Euo to its targets *in vitro*.

**Figure 7:**
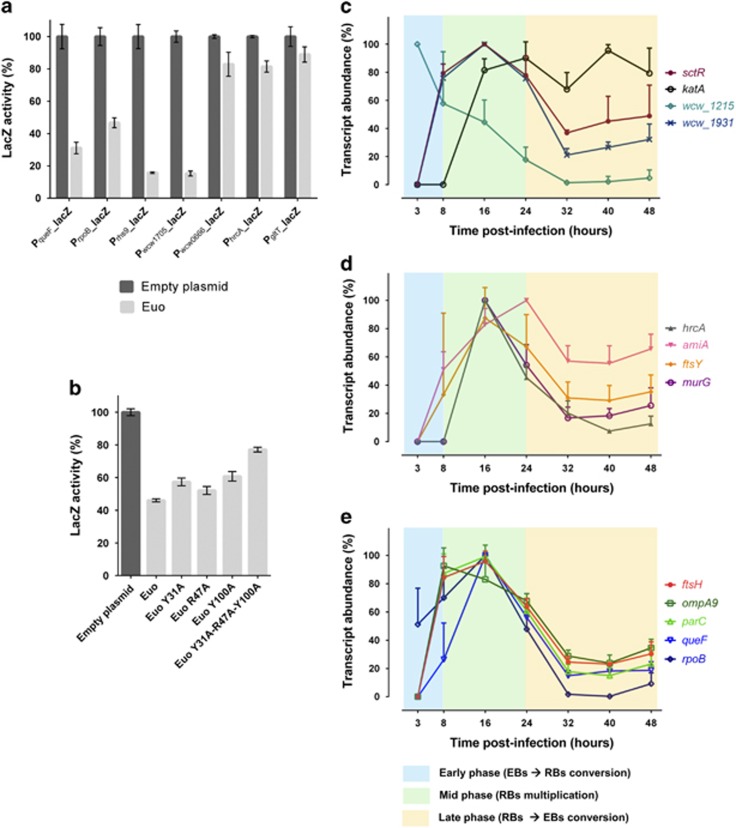
Developmental control by Euo. (**a**) LacZ-based promoter-probe plasmids were co-transformed into *E. coli* with a plasmid carrying *euo* under the control of an IPTG-inducible promoter (pSRK-Gm) (Khan *et al.,* 2008). As a control, we used an empty expression vector. The LacZ activity was determined and the data represent mean values±standard deviation of three biological triplicates. When Euo expression is induced, the LacZ activity strongly decreased for the P_*queF*_, P_*rpoB*_, P_*rhs9*_ and P_*wcw_1705*_ and slightly decreased for the P_*wcw_0066*_, P_*hrcA*_ and P_*gltT*_. (**b**) Transcriptional interference assays as in (**a**) with plasmids expressing the point mutant versions of Euo as indicated in the panel. Mutations are located in the two TorI-like wHTH arranged in tandem (individually and in combination) and cloned in a pBAD22 vector under the control of an arabinose-inducible promoter. These plasmids were co-transformed into *E. coli* with the plasmid carrying the P_queF_-*lacZ* fusion. Euo strongly decreased (by 60%) the LacZ activity for the P_*queF*_promoter, while the triple mutant only decreased the activity by 30%. (**c**–**e**) Temporal expression of Euo target genes was assessed by qRT-PCR at different time p.i. The transcript abundance (%) during the developmental cycle was determined for each Euo target gene. Data are mean values±standard deviation of three independent experiments. Most of the genes exhibited a peak of expression during the mid-phase of the developmental cycle and were considered as mid-phase genes. *Wcw_1215* is an early gene since the peak of transcript abundance was at 3 h p.i. whereas *katA* is a late gene since the expression remained stable during the late phase.

Structural predictions using HHpred (Soding *et al.*, 2005) revealed a resemblance of Euo to the DNA-binding domain of DNA excisionases such as Xis encoded in *Enterococcus faecalis* Tn916 or TorI from *E. coli* controlling recombination of the KlpE1 prophage (Elantak *et al.*, 2005). TorI folds into a winged helix-turn-helix (wHTH) (Elantak *et al.*, 2005), a DNA-binding motif comprising three alpha helices followed by 3–4 beta sheets (the wing), and assembles into stable dimers and multimers in the presence of target DNA. We also observed that the presence of DNA favours dimerization of His_6_-Euo *in vitro* (Supplementary Figure 7). Interestingly, the full-length Euo protein appears to harbour TorI-like wHTHs arranged in tandem, the first from residues 6–62 and the second from residues 78–137, suggesting that an Euo dimer can bind multiple minimal Euo boxes and that this tandem arrangement of putative wHTH promotes the formation of a stable nucleoprotein complex at chlamydial promoters, potentially accounting for the binding to the multiple minimal Euo boxes described above. We mutated the conserved arginine and tyrosine residues (individually and in combination) in the Euo wHTH that are required for DNA binding of TorI (Panis *et al.*, 2012) and found that the triple mutation (Y31A/R47A/Y100A) impaired binding of Euo on the P_*queF*_ promoter fused to the *lacZ* gene, in the *E. coli* transcription interference assay (Figure 7b). Thus, binding of Euo resembles that of TorI-like excisionases.

### Euo targets are developmentally regulated

As Euo was proposed to regulate late gene expression in *C. trachomatis* (Rosario and Tan, 2012; Rosario *et al.*, 2014), we explored whether this is also the case for *W. chondrophila* Euo, by determining the transcript profiles of selected Euo target genes during the *W. chondrophila* developmental cycle by qRT-PCR. The selected genes were from diverse functional categories, including stress adaptation (*hrcA[wcw_1636]*, *ftsH[wcw_0685], dps[wcw_0932]*), cell division and morphogenesis (*parC[wcw_1561]*, *sctR*, *amiA[wcw_0354]*, *murG[wcw_1386]*), general homeostasis/metabolism (*rpoB[wcw_0592], ftsY[wcw_0423]*, *gspE[wcw_1931], queF[wcw_0365], engA[wcw_1705]*) and unknown functions (*ompA9[wcw_1315]*, *wcw_1215)*. Almost all transcripts showed a mid-phase profile with a peak in abundance between 16 and 24 h p.i. (Figures 7c–e). Only the *wcw_1215* showed an early gene expression profile and the *katA* a late gene expression profile. Thus, the transcripts of Euo targets peak in mid-phase of the *W. chondrophila* developmental cycle.

Interestingly, *W. chondrophila* does not appear to target any genes orthologous to those identified as *in vitro* targets of Euo in *Chlamydia* (Zhang *et al.*, 1998, 2000; Rosario and Tan, 2012; Rosario *et al.*, 2014). We therefore investigated the differences of the *W. chondrophila* Euo regulon across the whole *Chlamydiales* order by BBH comparison. Clustering of similar sequences revealed the presence of two main groups: a small cluster including highly conserved (>50% identity) proteins across the *Chlamydiales* order, and a second large cluster of less conserved proteins (<40% identity) (Supplementary Figure 3). Even though this list does not include any genes targeted by *C. trachomatis* Euo *in vitro*, our list of *W. chondrophila in vivo* targets includes several conserved genes whose transcripts are developmentally regulated in *C. trachomatis* (for example, *amiA* (*ct2687*/*wcw_0354*), *hrcA* (*ct394/ wcw_1636*), *ftsI* (*ct682/wcw_1931*) and *sctR* (*ct562*/*wcw_0093*)).

## Discussion

The reduced regulatory genomes of obligate intracellular *Chlamydiae* offer a unique opportunity to explore which regulatory systems are dispensable for developmental control. C-di-GMP- and histidine kinase-based regulatory systems that are most commonly used for post-translational regulation in free-living bacteria (West and Stock, 2001; Hengge, 2009) are sparsely (if at all) encoded in chlamydial genomes. Additionally, as only 10 conserved (annotated) TFs (Figure 1) could control transcript abundance over the developmental cycle (Belland *et al.*, 2003; Albrecht *et al.*, 2010), we aimed to define the regulatory genome directing intracellular growth and/or differentiation in a system with low TF multiplicity offered by chlamydiae. Using ChIP-Seq, we unearthed the landscape of conserved chlamydial TF specificities and provided strong evidence that the chlamydial signature protein Euo acts as a master TF that controls developmental transcription. Thus, transcriptional reprogramming during the chlamydial developmental cycle is governed at least in part at the level of transcription initiation by TFs that selectively bind promoters of developmentally-regulated genes and are themselves under temporal control. The pervasive binding of Euo to 109 predicted target sites represents more than 5% of the potential transcriptome that could be influenced directly by this TF and possibly further magnified by (direct or indirect) transcriptional or post-transcriptional control systems (for example, by anti-termination or mRNA degradation, respectively).

The predicted number of direct Euo targets is well within the range for master TFs known from other developmental systems. For example, the α-proteobacterial master TF CtrA (Quon *et al.*, 1996), an extended member of the OmpR superfamily of DNA-binding response regulators, is also predicted to target >100 promoters in the free-living α-proteobacterium *Caulobacter crescentus* that regulates at least 20% of its transcriptome as a function of its developmental cycle and has a genome twice the size of that of *W. chondrophila* (Laub *et al.,* 2000, 2002; Nierman *et al.*, 2001; Fiebig *et al.*, 2014; Fumeaux *et al.*, 2014). Interestingly, CtrA acts as a master TF not only in free-living α-proteobacteria (Brilli *et al.*, 2010; De Nisco *et al.*, 2014; Fumeaux *et al.*, 2014; Panis *et al.*, 2015), but has recently been implicated in targeting several developmentally-regulated promoters in the α-proteobacterial intracellular pathogen *Ehrlichia chaffeensis* (Cheng *et al.*, 2011), the aetiological agent of tick-borne human ehrlichiosis. *E. chaffeensis* branches with the rickettsial lineage of obligate intracellular α-proteobacteria and exhibits a developmental cycle bearing remarkable similarity to that of chlamydia, with the dense-cored cells acting as infectious forms that enter the host, differentiate into reticulate cells that eventually morph into dense-cored cells (Zhang *et al.*, 2007), released from host cells. Thus, Euo may act as chlamydial counterpart of the α-proteobacterial master (developmental) regulator CtrA. Based on the distinct primary structures of the two proteins, mechanistic differences may clearly underlie the conceptual resemblance. Moreover, it was recently reported that Euo has been vertically inherited during chlamydial evolution strengthening the role of Euo as a master regulator (Domman and Horn, 2015). The overwhelming majority of Euo regulated genes encode mid-phase transcripts (Figure 7) that are required for the rapid proliferative (RB) phase in chlamydial development following the peak of Euo abundance in *W. chondrophila* (at 8 h p.i., Figure 2).

Speculating on the mode of binding of Euo to DNA via TorI-like wHTHs, it is noteworthy that multiple TorI binding sites are thought to have a crucial role in (i) bending DNA (nucleoprotein filament) and (ii) positioning recombination integrase proteins for proper formation of the excisive nucleoprotein complex (Panis *et al.*, 2010a, b, 2012). Thus, these Euo boxes could also serve in proper positioning of RNAP for open complex formation in transcription initiation. The association of Euo with transposase genes also resembles the repression of the integrase gene by TorI (Panis *et al.*, 2010b). Typically, the formation of higher order nucleoprotein complexes leads to repression while a lower order complex can promote transcriptional activation. Distinct promoter architectures of such minimal Euo boxes may dictate whether Euo acts as activator or repressor, of the many divergent Euo-targeted promoters.

It is certainly possible, or even likely, that the reprogramming of the chlamydial transcriptome is reinforced by other TFs acting (sequentially) in isolation or as modules (Niehus *et al.*, 2008; Vijayan *et al.,* 2009; Case *et al.*, 2010; Tan, 2012; Domman and Horn, 2015). Such strategies are known for α-proteobacteria (Panis *et al.*, 2015) directly at the transcriptional or indirectly at the post-transcriptional level for fine-tuning developmental regulation. Intriguingly, our ChIP-Seq data indicate that the Wcw_1223 TF that is encoded in the genomes of *Chlamydiae* and *Planctomycetes*, but has not yet been studied, functions as a master TF across the chlamydial and planctomycetes phyla. Wcw_1223 also targets >100 putative sites in *W. chondrophila* and like Euo controls genes of different functional categories. Moreover, using the Phyre2 prediction server (Kelley and Sternberg, 2009) we noted a structural similarity to the predicted transcriptional regulator VC0467 from *Vibrio cholerae*.

Akin to Euo, HrcA is not present in the *Lentisphaerae, Verrucomicrobia* and *Planctomycetes* (which seems to encode RpoH/σ^32^) (Wecker *et al.*, 2009), but is present throughout the phylum *Chlamydiae* (Figure 1) (Hecker *et al.*, 1996; Tan *et al.*, 1996; Narberhaus, 1999; Wilson and Tan, 2004; Hanson and Tan, 2015). This distribution begs the question whether HrcA has been appropriated for chlamydial development and/or pathogenesis. The *W. chondrophila* HrcA regulon is quite small (<5%) compared with that of Euo and includes *hrcA* as well as genes involved in the adaptive response to temperature upshift, such as the (co-) chaperonin encoding genes *groEL/ES*, *dnaK* and *grpE* (Domman and Horn, 2015). In *Chlamydia*, the regulation of *dnaK* and *groE* operons was of great interest because of their role in pathogenesis and because of their induction upon temperature upshift due to a decrease in HrcA binding on these promoters (Hanson and Tan, 2015). In this context, we note that a widely used concept to modify gene expression in pathogenic bacteria upon host cell entry is that of protein thermometers, changing the activity upon temperature upshift in the host compared with the lower temperature in the environment (Kamp and Higgins, 2011; Loh *et al.*, 2013) and HrcA is a thermosensor in the human pathogen *Helicobacter pylori* (Roncarati *et al.*, 2014). Moreover, some *Chlamydia*-related bacteria have been shown to be stable endosymbionts of amoebae when present in the environment at a temperature of ⩽30 °C but then exhibit a lytic phenotype towards amoebae when the temperature increased above 32 °C (Greub *et al.,* 2003). Thus, the pathogenic potential of *Parachlamydia* towards amoebae and higher eukaryotes including humans and bovines (Borel *et al.*, 2007; Greub, 2009) might be due to an activation of temperature-regulated genes. Interestingly, we also note that HrcA targets the promoter of a gene (*wcw_0325*) encoding a protein featuring a von Willebrand factor type A domain known to act as mechanosensors (Siryaporn *et al.*, 2014), possibly signalling the interaction of the bacterium with its target host cell upon temperature upshift in the host.

By contrast to such phylum-specific regulation, PhoB of the *Chlamydiaceae* (where it is called ChxR, Figure 1) is not very similar to that encoded in the other chlamydial families. Moreover, unlike PhoB, ChxR does not possess the conserved Asp residue and likely regulates genes expression independently of the activation by phosphorylation. With the putative PhoR-like histidine kinase Wcw_1870 following the same evolutionary pattern (that is, absence from the *Chlamydiaceae*), we suspect that PhoB may have become functionally specialized as the *Chlamydiaceae* branched from the ancestral lineage. In support of this, many of the promoters targeted by *W. chondrophila* PhoB appear to direct expression of hypothetical proteins, and none of the five known members of the *C. trachomatis* ChxR regulon (Koo *et al.*, 2006; Hickey *et al.*, 2011) were found to be regulated by PhoB *in vivo*. From an evolutionary perspective, it will be very interesting to define the PhoB regulon in other members of the PVC superphylum to determine whether orthologous genes are targeted by this TF. Indeed, PhoB of the widespread and highly diverse *Chlamydia*-related bacteria is more similar to PhoB of *Planctomycetes*, *Verrucomicrobia* and *Lentisphaerae* than to the ChxR homologue in *Chlamydiaceae,* suggesting that this TF may regulate genes implicated in the survival and in the colonization of a large variety of environmental ecosystems. This will be particularly interesting given the different ecology of the free-living versus obligate intracellular PVC superphylum members. Moreover, members of the *Verrucomicrobia* are important determinants of the human microbiome, and thus also have the capacity to interact (directly or indirectly) with eukaryotic host cells (Chevalier *et al.*, 2015).

## Figures and Tables

**Table 1:** Summary of the validation of Euo target sites from ChIP-Seq by EMSA

*Gene*	*Predicted function*	*Synonym*	*Shift*
*wcw_0093*	Flagellar biosynthesis pathway, component FliP	*sctR*	++++
*wcw_0203*	Histone H1-like protein Hc1	*hctA*	–
*wcw_0354*	*N*-acetylmuramoyl-l-alanine amidase	*amiA*	+++
*wcw_0365*	Enzyme related to GTP cyclohydrolase I	*queF*	++++
*wcw_0390*	Rhs family protein	*rhs9*	++++
*wcw_0423*	Signal recognition particle GTPase	*ftsY*	+++
*wcw_0430*	NADH dehydrogenase, FAD-containing subunit	*ndh*	++++
*wcw_0544*	Integrase	*wcw_0544*	–
*wcw_0547*	Transposase	*wcw_0547*	+
*wcw_0592*	DNA-directed RNA polymerase, beta subunit/140 kD subunit	*rpoB*	+++
*wcw_0655*	Catalase	*katA*	++++
*wcw_0685*	ATP-dependent Zn proteases	*ftsH*	+
*wcw_1215*	Outer membrane protein		+
*wcw_1315*	Hypothetical protein	*OmpA9*	++
*wcw_1386*	UDP-*N*-acetylglucosamine:LPS *N*-acetylglucosamine transferase	*murG*	+
*wcw_1561*	Type IIA topoisomerase (DNA gyrase/topo II, topoisomerase IV), A subunit	*parC*	++
*wcw_1636*	Transcriptional regulator of heat shock gene	*hrcA*	++++
*wcw_1705*	Predicted ATPase or kinase	*wcw_1705*	+++
*wcw_1731*	Na+/H+-dicarboxylate symporters	*gltT*	++++
*wcw_1924*	Secreted protein		++++
*wcw_1931*	Cell division protein FtsI/penicillin-binding protein 2	*ftsI*	+++

Abbreviations: ChIP-Seq, chromatin-immunoprecipitation-sequencing; EMSA, electrophoretic mobility shift assay. ++++ for completely shifted at 1800 nm; +++ for shifted band bigger than the PCR band at 1800 nm; ++ for shifted band appears at 240 nm; +for shifted band appears at 960 nm; − for no shifted band observed.

List of target promoters used as probes to test for binding by His_6_-Euo by EMSA. The number of plus symbols indicates the efficiency of binding.
