# Supplementary material for: Regulatory (pan-)genome of an obligate intracellular pathogen in the PVC superphylum
Source: ISME J. 2016 Mar 8;10(9):2129–44. doi: 10.1038/ismej.2016.23 (PMC4989314; doi:10.1038/ismej.2016.23)
Supplement: Supplementary Information [file ismej201623x1.doc]

**Supplementary Information**


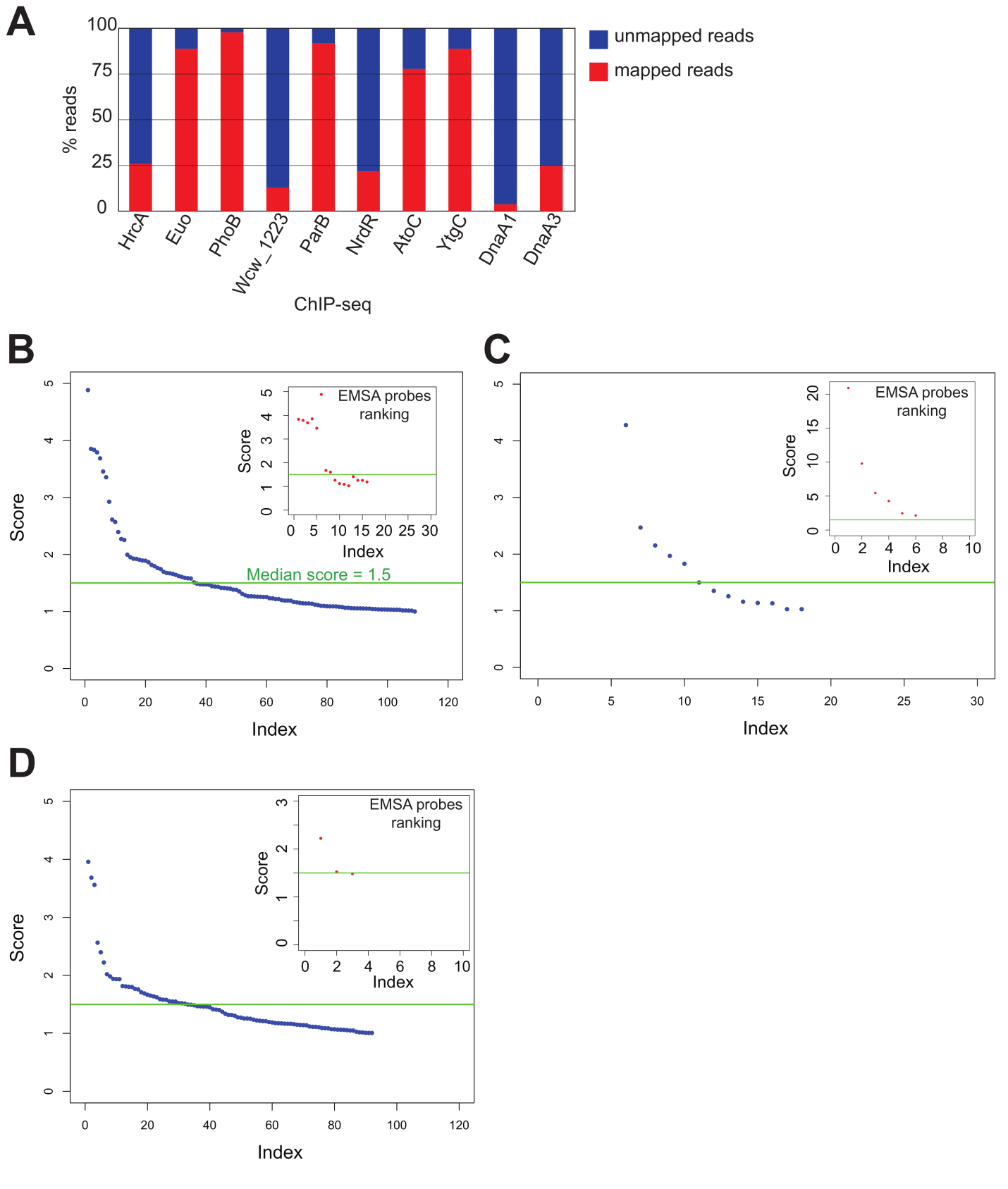
**Figures**

Supplementary Figure 1. ChIP-Seq analysis of the ten *W. chondrophila* TFs. (A) Bowtie alignment statistics depicting the percentage of mapped and unmapped reads on the genome of *Waddlia chondrophila*. (B-D) Ranking of the Euo (B), HrcA (C) and PhoB (D) total predicted targets, based on the percentage of reads per probe, included in the graph is reported also the ranking of the predicted targets used for *in vitro* and *in vivo* assays.

**
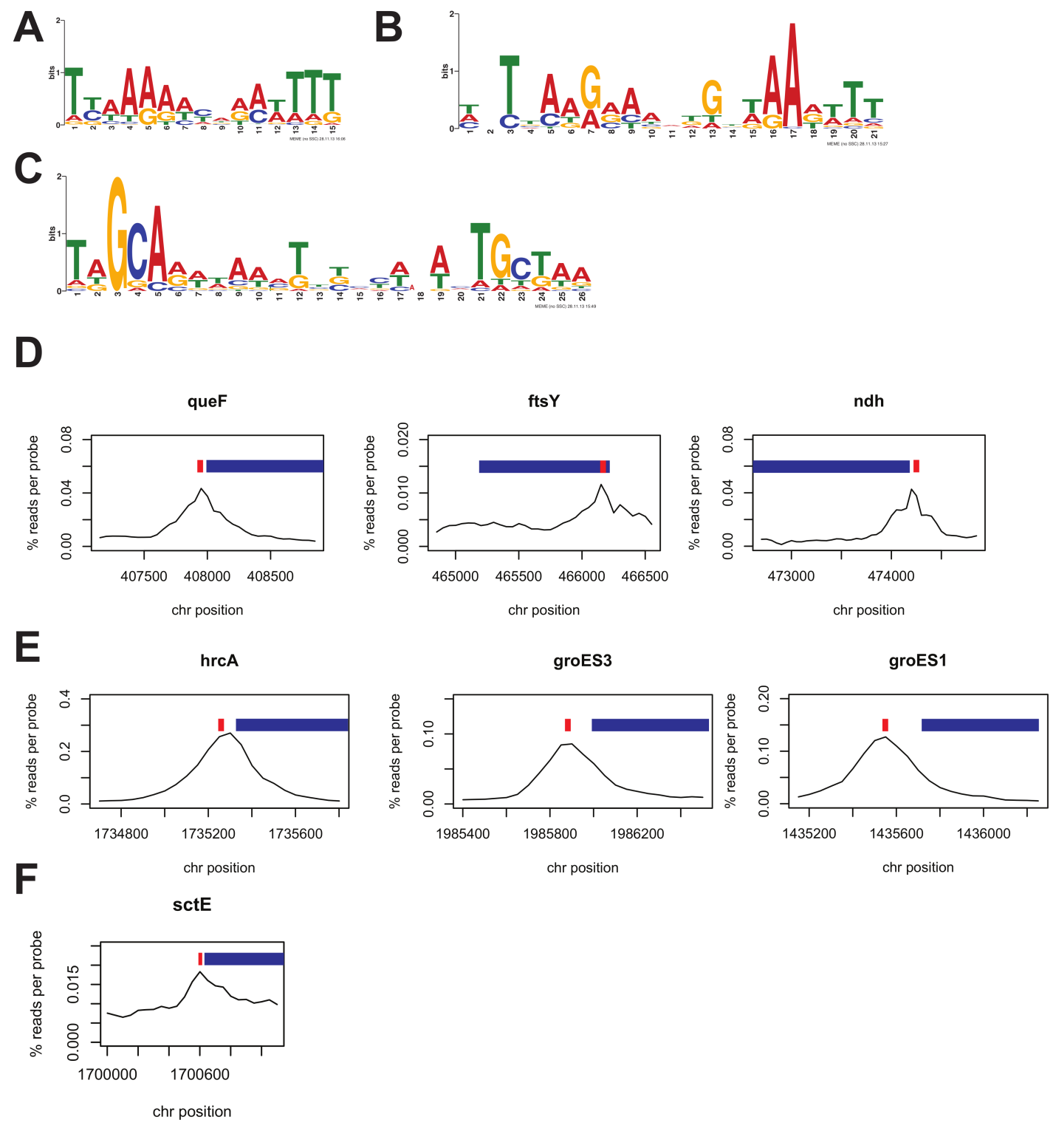
**

Supplementary Figure 2. Motif and positional analysis of the ChIP-Seq data. (A-C) *W. chondrophila* consensus motif for Euo (A), PhoB (B) and HrcA (C) as identified by MEME *de novo* motif discovery. (D) ChIP-Seq profiles of the region encompassing *queF*, *ftsY* and *ndh* genes used in EMSAs and for *E. coli* transcriptional interference assays. Location of the Euo consensus (red) with respect to the gene putatively regulated (blue) are reported in figure. (E) ChIP-Seq profiles of the region encompassing *hrcA*, *groES3* and *groEL1* genes used in EMSAs and for *E. coli* transcriptional interference assays. Location of the HrcA consensus (red) with respect to the gene putatively regulated (blue) are reported in figure. (F) ChIP-Seq profiles of the region encompassing *sctE* gene. Location of the PhoB consensus (red) with respect to the gene putatively regulated in blue in the figure.


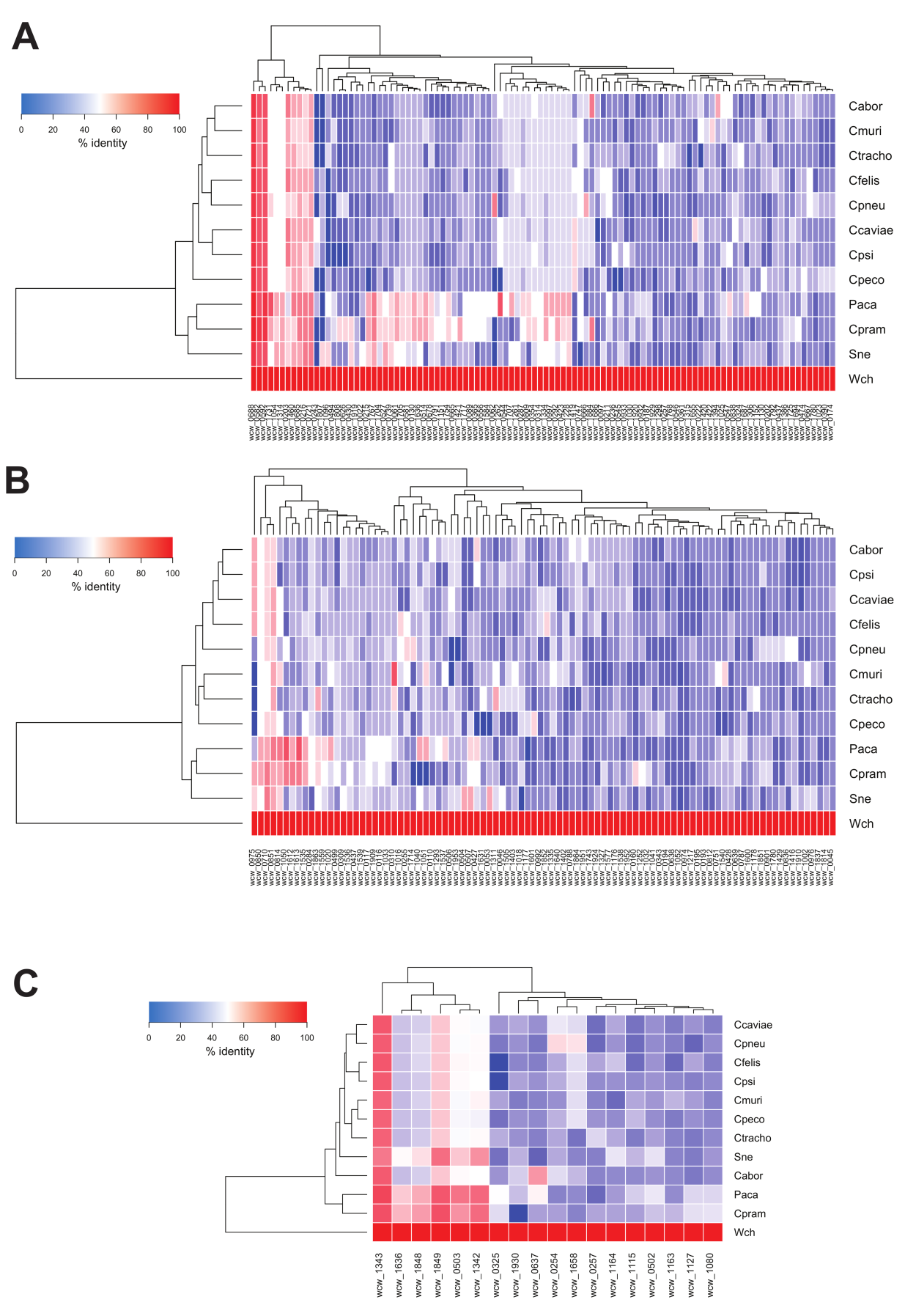


Supplementary Figure 3. Conservation of regulons across the chlamydial phylum. Panels showing the conserved regulon of Euo (A), PhoB (B) and HrcA (C) in 13 *Chlamydiales* genomes based bidirectional best blast hit (BBH) analysis compared to the *W. chondrophila* orthologs.

**
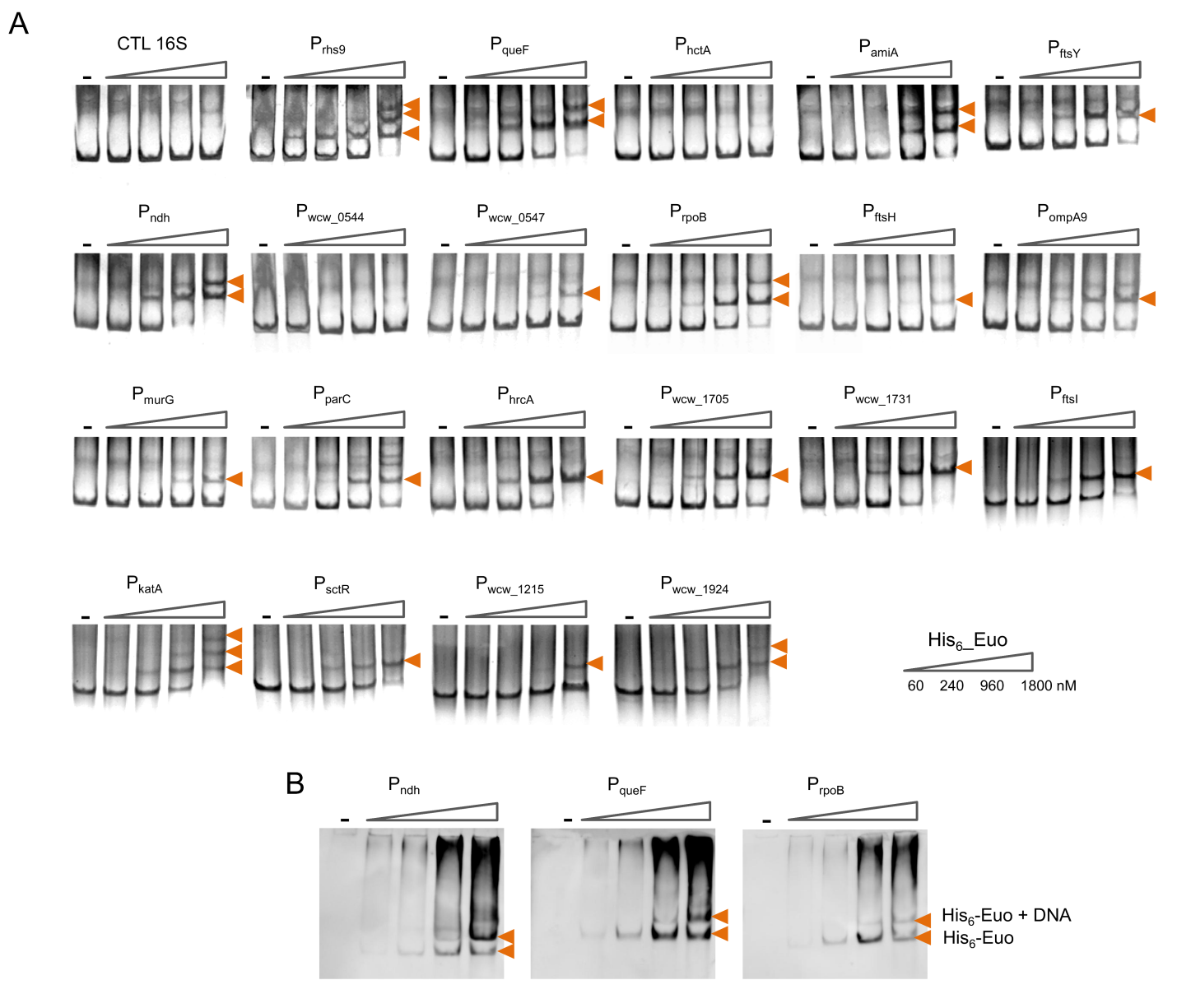
**

Supplementary Figure 4. Validation of Euo targets by EMSA. (A) Twenty-one promoters were amplified by PCR and used for EMSA. Eighty ng of DNA fragments were incubated in absence (-) or in presence of an increasing concentration of His_6_-Euo. The DNA was detected by GelRed. As a negative control, we used a specific PCR targeted the 16S rRNA gene. Shifted band were observed for all DNA fragments except the negative control, the P*_hctA_* and the P*wcw_0544*. (B) Binding of His_6_-Euo on the P*_ndh_*, P*_queF_* and P*_rpoB_*. Euo alone or in complex with DNA was detected by immunoblot using specific mouse polyclonal anti-Euo antibody. Protein-DNA complexes were observed for the 3 promoters.


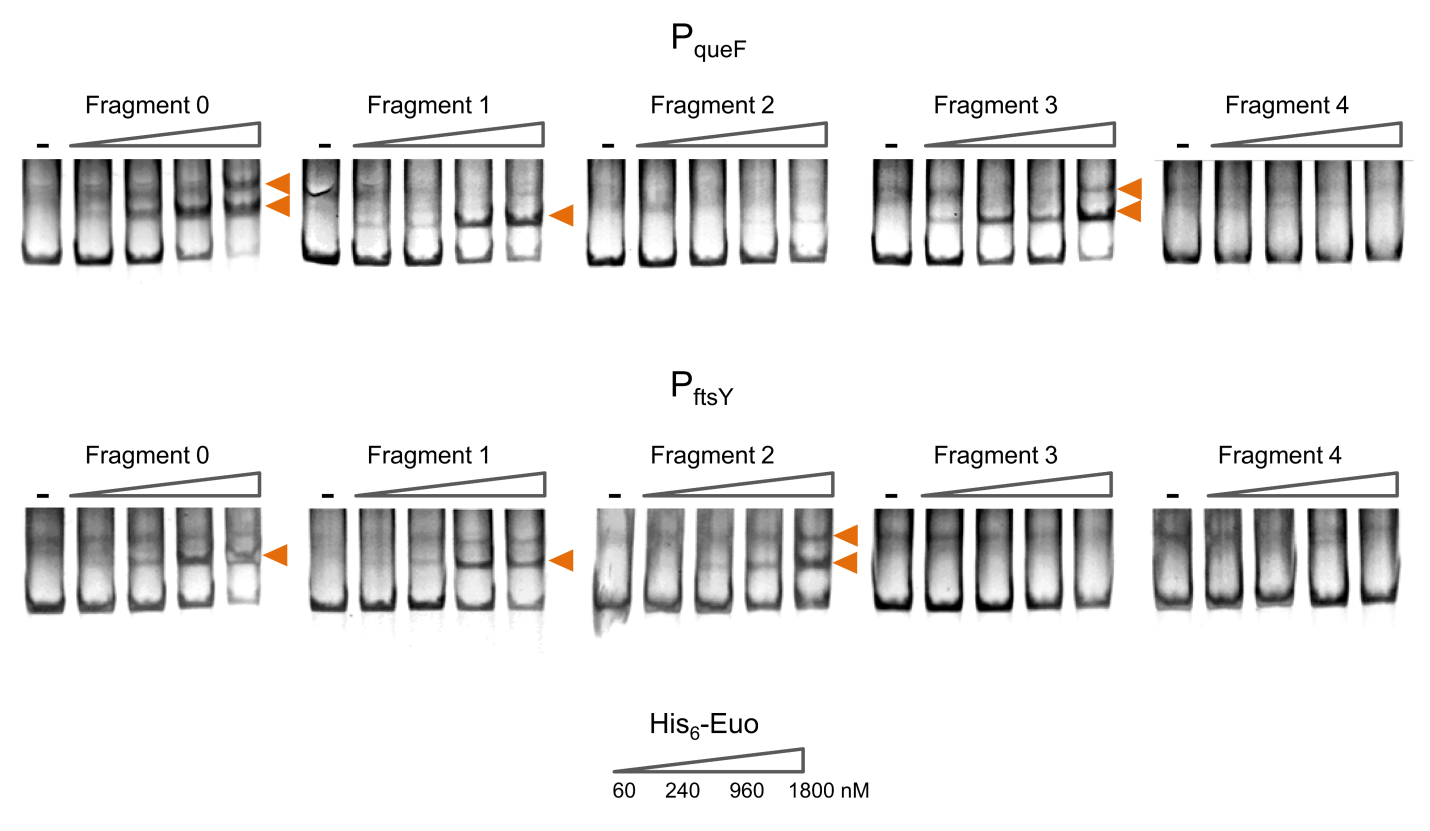


Supplementary Figure 5. Identification of a 50-bp region necessary for the binding of His_6_-Euo which includes the consensus. PCRs shifted by 50 bp were designed for the P_queF_ and P_ftsY_ promoters and all fragments were used for *in vitro* binding assay (EMSA) with His_6_-Euo. A total of 80 ng of DNA fragments were incubated in absence (-) or in presence of an increasing concentration of His_6_-Euo. Protein-DNA complexes were detected using GelRed. No shifted bands were observed for fragments 2 and 4 of P*_queF_* and for fragments 3 and 4 of the P*_ftsY_*.


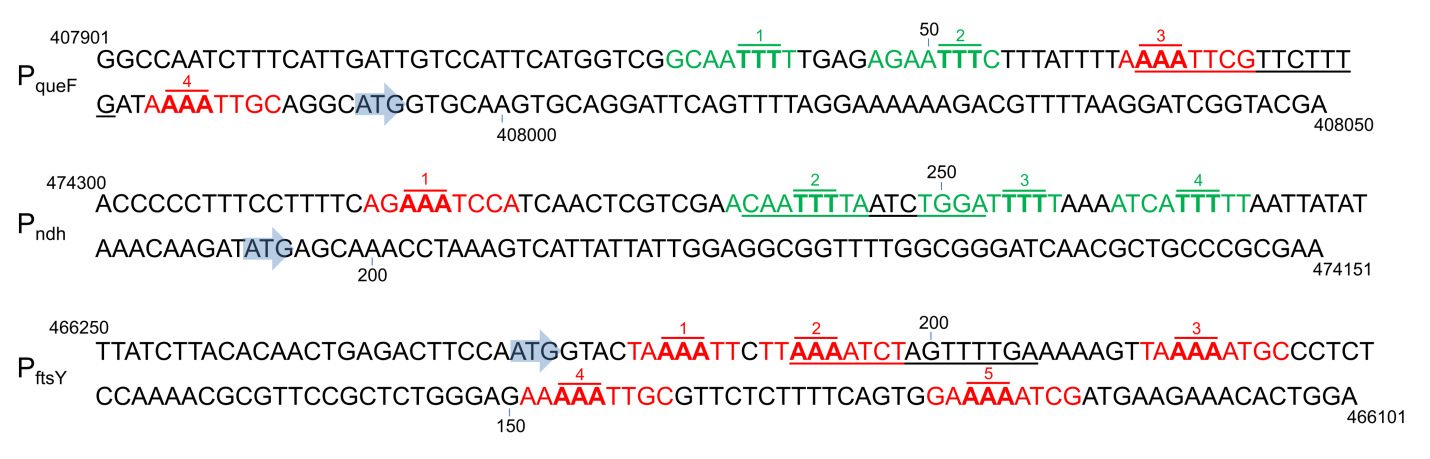


Supplementary Figure 6. Identification of a Euo-box present in the P*_queF_*, P*_ndh_* and P*_ftsY_* promoters. Euo-boxes are depicted in green (+ strand) or in red (- strand). The conserved TTT present in the Euo-boxes were underlined. The arrow indicates the start codon. The Euo consensus binding site, based on the ChIP-Seq results, is also underlined. In each promoter, the conserved TTT of one minimal Euo-box is present in the Euo consensus binding site.


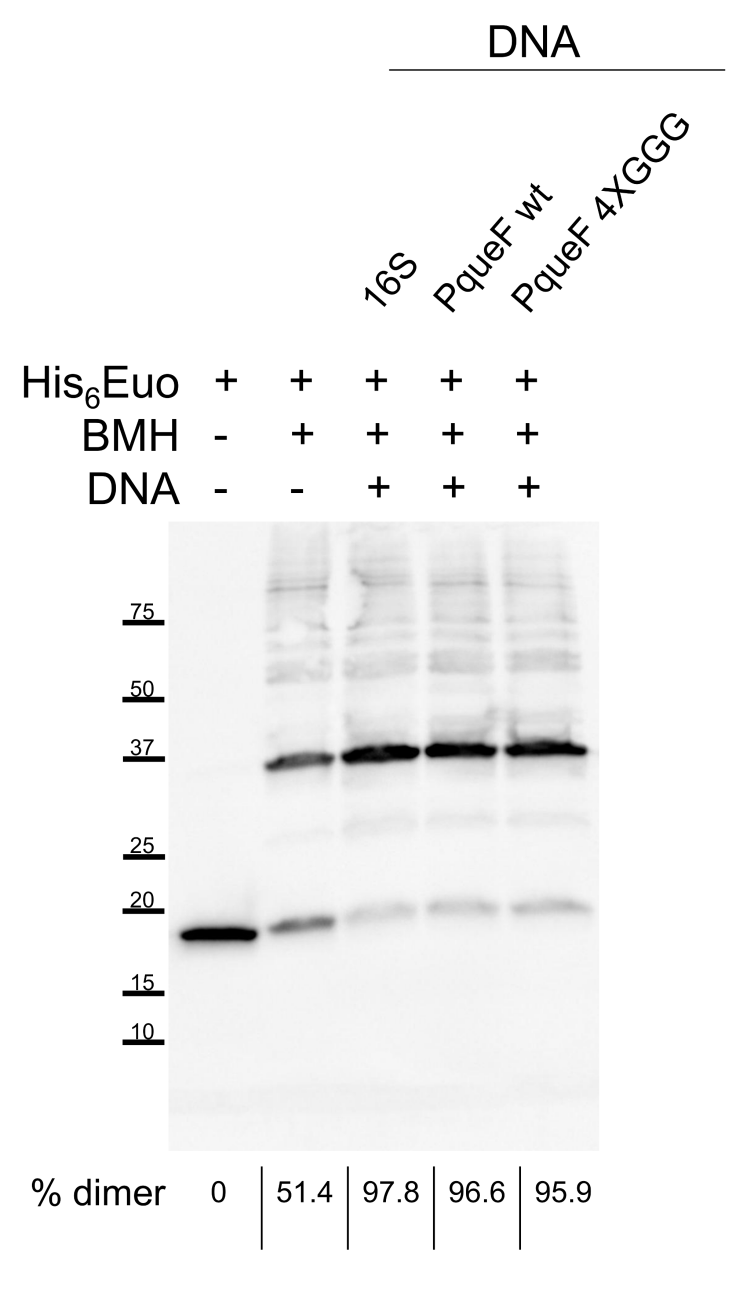


Supplementary Figure 7. Euo dimerizes in presence of DNA. Five μM of His_6_-Euo was incubated in presence or absence of 1 mM of BMH, a crosslinking agent. When indicated, 80 ng of PCR fragment were added to the reaction. Samples were separated by SDS-PAGE and His_6_-Euo detected by immunobloting using mouse monoclonal anti- His_6_  antibodies.

**Supplementary Table Legends**

**Supplementary Table 1. The ChIP-Seq data for the 10 TFs.** Each sheet corresponds to ChIP-Seq data set of one TF and lists the peaks (binding sites) near annotated genes.

**Supplementary Table 2. The 18 HrcA target sites and the motif associated to them. Column A**. Gene, name of the gene associated to the probe identified in the ChIP-seq experiment. **Column B**. PID, PubMed Identification number. **Column C**. %reads, abundance measurements of the ChIP signal for the probe took into consideration. **Column D**. Score, median score. **Column E**. Probe start, genomic coordinate of the 50bp probe. **Column F**. Probe end, genomic coordinate of the 50bp probe. **Column G**. ORF orientation, genome orientation of the gene associated to the probe. **Column H**. ORF start, genomic coordinate of the gene associated to the probe. **Column I**. ORF end, genomic coordinate of the gene associated to the probe. **Column J**. motif dist ATG, distance (in nucleotides) of the consensus motif from the ATG of the gene associated to it. **Column K**. Motif E- value, E-value of the consensus motif. **Column L**. Motif sequence. **Column M**. Name, common name of the gene. **Column N**. predicted function, putative function of the gene.

**Supplementary Table 3. The 44 PhoB target sites and the motif associated to them.**

**Supplementary Table 4. The 109 Euo target sites and the motif associated to them.**

**Supplementary Table 5. Primers used for the RT-qPCR.**

**Supplementary Table 6. Primers used for the shift assay.**

**Supplementary Table 7. Synthetic DNA used for the shift assay.**

**Supplementary Table 8. Detailed ChIP-Seq information**. ChIP sample: Name of the ChIP-seq sample as in Supplementary Table 1. Library Name: Name of the ChIP-Seq library. Anti-serum: Antibodies used in the ChIP experiment. Hpi: time of sampling, in hours post infection, EBs, elementary bodies post purification. Serum: Origin of the anti-serum M, mice, R, rabbit, pM, pre-immune serum mice, pR, pre-immune serum rabbit. Tot Reads number: total number of reads after deep sequencing. Aligned reads: Number of reads with one reported alignment. Lost reads: Reads that failed to align. Percentage of aligned reads: Number of reads with one reported alignment reported as a percentage of the total number of reads. Percentage of unmapped reads: Number of reads that fail to align reported as a percentage of the total number of reads.

**Supplementary Table 9. *Waddlia chondrophila* proteins used as queries for phylogenetic genome analysis.**

**SUPPLEMENTARY METHODS**

**Plasmid construction, Proteins purification and antibodies production**

The His_6_- overexpression plasmid for each of the ten conserved TFs were made by amplifying each TF gene [as annotated by the *Waddlia chondrophila* genome sequence (NC_014225)], after digestion with *Nde*I/*Eco*RI (or *Mun*I in cases where am internal *Eco*RI site was present) into pET28a encoding the His_6_- tag (EMD Biosciences, La Jolla, CA) or into the cloning vector pMT335 (Radhakrishnan et al 2010, Thanbichler et al 2007). Restriction digest with *Nde*I/*Sac*I or *Nde*I/*Xba* needed to ligate the fragments in the pET28a-derivative pCWR547 encoding the His_6_-SUMO tag (Radhakrishnan et al 2010) or SRK-Gm (Khan et al 2008). The *Nde*I recognition sequence overlapped the start codon while the *Eco*RI (*Mun*I) sequence overlapped the stop codon. For cloning into pBAD22 (Guzman et al 1995), an *Nco*I cloning strategy was used in which the *Nco*I recognition sequence overlaps the start codon and part of the second codon (set to encode serine).

Overexpression constructs for each of the ten conserved chlamydial TFs were expressed either from pET28a or pCWR547 in *E. coli* Rosetta (DE3)/pLysS (EMD Biosciences, La Jolla, CA) and purified under native conditions (Euo, NrdR, HrcA, ParB, PhoB) using Ni^2+^ chelate chromatography. A 5mL overnight culture was diluted into 1 L of pre-warmed LB. OD_600nm_ were monitored until OD_600nm_ =~ 0.3-0.4, then 1mM IPTG was added to the culture and growth continued. After 3 hours cells were pelleted, and re-suspended in 25 mL of lysis buffer (10 mM Tris HCl pH8, 0.1 M NaCl, 1.0 mM β-mercaptoethanol, 5% glycerol, 0.5 mM imidazole triton 0.02%). Cells were sonicated (Sonifier Cell Disruptor B-30; Branson Sonic Power*.* Co., Danbury, CT) on ice using 12 bursts of 20 seconds at output level 5.5. After centrifugation at 6’000 rpm for 20 minutes, the supernatant was loaded onto a column containing 5 mL of Ni-NTA agarose resin pre-equilibrated with lysis buffer 1X. Column was rinsed with lysis buffer, 400 mM NaCl and 10 mM imidazole, both prepared in lysis buffer. Fractions were collected (in 300 mM Imidazole buffer, prepared in lysis buffer 1X) and resolved on a 12.5 % SDS polyacrylamide gel. Purified fractions were used to raise polyclonal antibodies in rabbits (Josman LLC, Napa, CA) and mice (Eurogentech, Searing, Belgium).

Purification under denaturing conditions (Wcw_1223, AtoC, YtgC, DnaA1, DnaA3) was performed as follow: pET28a or pCWR547 derivatives for each of the ten TFs were expressed in *E. coli* Rosetta (DE3)/pLysS and purified under denaturing conditions (in 8 M Urea, NaH_2_PO_4_ 100mM, Tris 25mM). A 5mL overnight culture was diluted into 1L of pre-warmed LB. OD_600nm_ were monitored until OD_600nm_ =~ 0.3-0.4, then 1 mM IPTG were added to the culture transferred at RT for 5 hours. After 5h induction cells were pelleted, re-suspended in 25 mL of lysis buffer (10 mM Tris HCl pH8, 0.1 M NaCl, 1.0 mM β-mercaptoethanol, 5% glycerol, 0.5 mM imidazole, triton 0.02%). Cells were sonicated (Sonifier Cell Disruptor B-30; Branson Sonic Power*.* Co., Danbury, CT) on ice using 12 bursts of 20 seconds at output level 5.5. After centrifugation at 6’000 rpm for 20 minutes, the supernatant were discarded and the pellet re-suspended in 25 mL of Buffer B (pH 8.0), then centrifuged at 6’000 rpm for 20 minutes, the supernatant was loaded onto a column containing 5 mL of Ni-NTA agarose resin. Column was rinsed with Buffer B, Buffer C (pH 6.3) and eluted with Buffer E (pH 4.5). Fractions were collected and resolved on a 12.5% SDS polyacrylamide gel. The protein was excised from a 15% SDS polyacrylamide gel and used to raise polyclonal antibodies in rabbits (Josman LLC, Napa, CA).

**qRT-PCR**

Vero (2.5x10^6^) cells were seeded in 25cm^2^ flasks and infected as described before. At different time post-infection, infected cells were washed once with PBS and 2.5 mL of TRIzol was added. Cells were scrapped and frozen at -80°C. RNA was extracted according to the manufacturer instructions. RNA was re-suspended in 50 μL of water and then treated with DNase, using the Ambion DNA-free kit (Life technologies, Grand Island, NY) to eliminate possible DNA contamination. cDNA was synthesized by reverse transcription using the GoScript Reverse Transcription system (Promega, Fishburg, WI). Random primers were used. The qPCR mixtures included 300 nM of each reverse and forward primers (Supplementary Table 5), 10 μL of iTaq universal SYBR green mix (BioRad, Hercules, CA) and 4 μL of cDNA. The qPCRs were performed on the Step One PCR system (Applied Biosystems, Zug, Switzerland) using the following conditions: 3 minutes at 95°C, 45 cycles of 15 seconds at 95°C and 1 minute at 60°C. A melting curve was then performed (15 seconds at 95°C, 1 minute at 55°C, +0.5°C increment, 15 seconds at 95°C). The amplification efficiency of each couple of primers, used at 300 nM, was determined and considered as good if comprised between 90 and 110%. The 16S rRNA encoding gene was used as the endogenous control and the 48h time point as the reference sample (Croxatto et al 2013). The fold change was calculated using the formula 2-^ΔΔCt^. To compare all expression profiles, for each gene, the maximum value was normalized to 100% and the minimal value to 0%. Experiments were done in triplicate; means and standard deviations were represented on the graphs.

**Immunoblotting**

For the expression profile of the TFs, Vero cells were infected in 25 cm^2^ flask as described above. At different time post-infection, infected cells were recovered after cell scrapping. One hundred μL were removed to proceed to genomic DNA extraction. The infected cells were centrifuged at maximal speed during 5 minutes. The pellets were washed once with PBS and then re-suspended in 500 μL of loading buffer (60mM Tris pH 6.8, 1% SDS, 1% mercaptoethanol, 10% glycerol, 0.02% bromophenol blue). Ten μL of each sample were separated by SDS-PAGE on a 12% polyacrylamide gel (BioRad, Reinach, Switzerland) and transferred on nitrocellulose membrane. Membranes were blocked during 2 hours in TBST (Tris base 10 mM, NaCl 150 mM, Tween 0.05%) with 5% of non-fat powder milk. The membranes were then incubated overnight at 4°C with the primary antibody (diluted in TBST with 0.5% of milk), washed three times with milk-TBST, incubated 2 hours with the secondary antibody conjugated to HRP (diluted 1/3000 in milk-TBST), washed three times with TBST and finally were detected by chemoluminescence using ImageQuant LAS 4000 Mini imager (GE healthcare, Waukesha, WI). Signals were quantified using ImageJ and normalized according to the number of bacteria per well. For each TF, the maximum value was then normalized to 100% and the minimal value to 0%. Experiments were done in duplicate. Polyclonal mouse antibodies to NrdR, DnaA1, PhoB, ParB, HrcA, Euo, YtgC and AtoC and rabbit polyclonal antibodies to Wcw1223 were used for immunoblotting.

**Chromatin Immuno-precipitation followed by deep sequencing (ChIP-Seq)**

ChIP-Seq was performed on infected Vero cells at different time p.i. as well as on purified EBs after amoebal co-culture using a sucrose barrier and a gastrografin gradient, as previously described (Greub et al 2003) (Supplementary Table 8). Vero cells were infected in 25 cm^2^ flasks as described above (10 flasks per ChIP-Seq for late time points, i.e. 60h and 72h p.i.; 40 flasks for early time point, i.e. 8h and 24h p.i.). At different time post-infection, infected cells were transferred in falcons and cross-linked in presence of 1% of formaldehyde and 10 μM of sodium phosphate (pH 7.6) during 10 minutes at room temperature followed by 30 minutes on ice (Fumeaux et al 2014). Purified EBs were washed twice with PBS and cross-linked as described for infected Vero cells. Cells were washed twice in phosphate buffer saline, re-suspended in TES buffer (10 mM Tris-HCl pH 7.5, 1 mM EDTA, 100 mM NaCl) containing 10 mM of DTT and incubated during 10 minutes at 37°C. Cells were pelleted and re-suspended in a Ready-Lyse lysozyme solution (Epicentre, Madison, WI), according to the manufacturer's instructions. The lysates were sonicated on ice by 10 cycles of 20 seconds to shear DNA and obtain fragments of length ranging from 300 to 700 bp. Samples were centrifuged at 14 000 rpm 2 minutes and the supernatants were take off and diluted to 1 mL in ChIP buffer (0.01% SDS, 1.1% Triton X-100, 1.2 mM EDTA, 16.7 mM Tris-HCl pH 8.1, 167 mM NaCl, containing protease inhibitors (Roche, Rotkreuz, Switzerland)). The diluted supernatants were pre-cleared with 150 μL of protein-A agarose (for rabbit polyclonal antibodies) protein-G agarose (for mouse polyclonal antibodies) (Roche, Rotkreuz, Switzerland). The pre-cleared supernatants were incubated overnight at 4° with 2 μL of specific polyclonal rabbit/mouse antibodies. The immuno-complexes were captured after incubation with Protein-A/G agarose pre-saturated with BSA during 2 hours at 4°C and washed once with low salt washing buffer (0.1% SDS, 1% Triton X-100, 2 mM EDTA, 20 mM Tris-HCl pH 8.1, 150 mM NaCl), then once with high salt washing buffer (0.1% SDS, 1% Triton X-100, 2 mM EDTA, 20 mM Tris-HCl pH 8.1, 500 mM NaCl), once with LiCl washing buffer (0.25 M LiCl, 1% NP-40, 1% deoxycholate, 1 mM EDTA, 10 mM Tris-HCl pH 8.1) and twice with TE buffer (10 mM Tris-HCl pH 8.1, 1 mM EDTA). The complexes were eluted twice with 250 μL of elution buffer (SDS 1%, 0.1 M NaHCO_3_, freshly prepared) and incubated overnight at 65°C with 300 mM NaCl to reverse the crosslinks. The samples were then treated with 2 µg of Proteinase K for 2 hours at 45°C in 40 mM EDTA and 40 mM Tris-HCl (pH 6.5). DNA was extracted using phenol:chloroform:isoamyl alcohol (25:24:1), ethanol-precipitated using 20 µg of glycogen as carrier and finally re-suspended in 100 μL of water. We conducted a first ChIP-Seq experiment with antibodies to the TFs at late time p.i., in order to obtain a high ratio of bacteria versus host cells. For TFs more abundant during the late phase of the developmental cycle (all except Euo and DnaA1), we decided to also perform ChIP-Seq on purified EBs in order to get rid of host cell DNA. Concerning Euo and DnaA1, which exhibit a different pattern of expression, we also did the ChIP-Seq at 8 and 24h, respectively. Further analyses were done on ChIP-Seq data set showing less background. As expected, ChIP-Seq done on purified EBs resulted in only about 10-25% of unmapped reads (PhoB, ParB, AtoC and YtgC) (Supplementary Figure 1), simplifying the identification of enriched genomic regions (see below and Supplementary Figure 2A-C).

**ChIP-Seq analysis**

Immunoprecipitated chromatin (*see chromatin immunoprecipitation for details*) was used to prepare sample libraries used for deep-sequencing at Fasteris SA (Geneva, Switzerland). ChIP-Seq libraries were prepared using the DNA Sample Prep Kit (Illumina) following manufacturer instructions (Fumeaux et al 2014). Single-end run were performed on an Illumina Genome Analyzer IIx or HiSeq2000, 50 cycles were read and yielded several million reads.

The single-end sequence reads stored in FastQ files were mapped against the genome of *Waddlia chondrophila* (NC_014225) using Bowtie (http://bowtie-bio.sourceforge.net/) allowing to map reads that match only one place in the genome (option used -q -m 1 -S). Bowtie results were converted into SAM/BAM formats by using samtools (<http://samtools.sourceforge.net/>). During mapping the reads, we observed a high percentage of unmapped reads (up to 95% of total reads, Supplementary Figure 1) stemming from contaminating host chromatin, especially in the samples processed with the antibodies to HrcA, Wcw_1223, DnaA1 and DnaA3. Nevertheless, we were still able to identify enriched genomic sites bound by these TFs (Supplementary Figure 2A-C, Supplementary Table 1). By contrast, others yielded only about 10% of unmapped reads, simplifying the identification of enriched genomic regions (Supplementary Figure 2A-C).

The standard genomic position format files (BAM) were imported into SeqMonk (<http://www.bioinformatics.babraham.ac.uk/projects/seqmonk/>, version 0.29.0) to build sequence read profiles. The initial quantification of the sequencing data was done in SeqMonk: the genome was subdivided into 50 bp probes, and for every probe we calculated a value that represents the number of reads which occur within the probe (using the Read Count Quantitation option). The quantitated probe list representing the genome was exported and used by a custom Perl script to compute the relative abundance for each probe in the genome from the overall reads count of each ChIP-Seq experiment(Fumeaux et al 2014). The script also calculated the average reads count and the standard deviation of the sample that are needed to establish a cut-off value in order to discern candidate peaks from background signal. In all the cases the average reads count plus twice the standard deviation of the sample was found to be effective to discern between candidate peaks and background noise. Peaks present in all samples (and in at least one ChIP-Seq experiment done with pre-immune sera to Wcw_1223 and Euo, Supplementary Table 8) were deemed sequencing artefacts and therefore removed manually. In order to identify high confidence target sites we computed a median score that is calculated only on the total number of targets. The median score is calculated taking into account only the probes that made the 2SD cut-off described above. Briefly, to quantify by how much each target is above the 2SD cut-off value, we calculated the ratio between the read percentage *per* probe of each ChIP-Seq target and the 2SD cut-off value. The median of the ratio is calculated and used to identify high confidence targets for each ChIP-Seq dataset. We choose to label as high confidence targets those having >1.5 median score.

*Mis-annotation corrections*

As sometimes mis-annotation errors (e.g. a peak is often annotated to the gene that is closer to it without considering the orientation of such gene) of the probes were found in the SeqMonk annotation report, in collaboration with Dr. Matteo Brilli (Edmund Mach Foundation, Trento, IT) we have developed an in-house Java (http://www.oracle.com/) program in order to re-annotate the probes to the correct gene (Fumeaux et al 2014). Our program scans the probes list of candidate peaks, and for each probe in the list, the distance from its centre to the start codon of nearby genes was calculated. A list of newly annotated probes having the maximum enrichment value in the peak, and a distance not greater than -500 and +100 from the start codon of the nearby gene(s) is generated. The new list of probes was used to retrieve 150 bp sequences centred on the probe plus the two adjacent 50 bp probes on each side for consensus motif predictions. In addition, in order to improve these predictions intergenic sequences as a seventh-order Markov model calculated using the intergenic sequences extracted from the two genomes.

The program resolves three possible annotation errors created by SeqMonk. When the probe is positioned in the intergenic region of two adjacent genes on the same DNA strand, SeqMonk often associates the probe to the closest gene without considering its orientation. In this case, the program does not consider the gene(s) upstream the selected probe and assigns the probe to the downstream gene if the distance from the centre of the probe is not greater than 500 bp (Bailey and Elkan 1995, Bailey et al 2006). In the second scenario, a probe might lie in the intergenic region of two divergent genes (e.g. the genes are on different DNA strands). In this case, the program assigns the probe to the gene when the distance to the start codon does not exceed 500 bp. In case both genes are less than 500 bp, the probe is assigned to both genes because no predictions can be made on which gene the binding site regulates. Finally, when the probe lies within the coding sequence of a gene, the probe is assigned to this gene, provided that the distance from the probe is less than 100 bp from the start codon. If the distance exceeds 100 bp, the probe is assigned to a nearby gene provided that the aforementioned criteria are fulfilled. All probes that fulfil these criteria are given association(s) to coding genes, but the “ANNO” list in the supplementary data reports only the probe with the highest relative abundance. Probes that cannot be linked to a coding gene are placed in a separate list (“NOANNO” in the supplementary data). The XLS files can also been deposited in the GEO database (accession number GSE68059).

*Consensus motif predictions*

The MEME (Bailey and Elkan 1995, Bailey et al 2006) motif finding tool were run on the set of 150 bp sequences retrieved from the *W. chondrophila* genome in order to identify the most over-represented motif for a given TF. In computational searches for *W. chondrophila* TF motif boxes, the likelihood that each sequence contained a box was compared with the likelihood that it was a typical *W. chondrophila* intergenic sequence (represented using a seventh-order Markov model).

For the Euo and HrcA ChIP-seq dataset, MEME runs were launched on the 109 and 20 predicted target sequences that made the cut-off described above. After MEME runs we retained only motifs with an *E*-value of ≥1.00•*e*^-05^. These sequences were used to build the PSSM describing the consensus motif and for generate the Sequence logo (http://weblogo.berkeley.edu/). The list of genes containing putative Euo consensus box were used to select those used in LacZ reporter assays or EMSAs.

Consensus motif prediction on the PhoB ChIP-seq dataset was made on the 46 high confidence targets. Six sites having an *E*-value of ≥1.00•*e*^-05^were cloned in plac290 and promoter activity tested.

*Ortholog identification*

In order to identify orthologs we used the identified TFs targets as queries of a dataset comprising completely sequenced genomes of thirteen organisms belonging to the *Chlamydiales*. Orthologous genes were identified with the bidirectional best blast hit method: the association of gene *a* in genome *X* and gene *b* in genome *Y* is known as bidirectional best hit, when *a* is the best hit of query *b* against all genes in *X* and *vice versa*. We used a 0.0001 e-value threshold for Blast analysis.

**Phylogenetic reconstruction**

First, we identified a list of 158 genes encoding proteins (Supplementary Table S9) used for homologous protein sequences were identified using orthoMCL version 2.0.9 (Li, Stoeckert, & Roos, 2003) with percentMatchCutoff=50, evalueExponentCutoff=-5 and an inflation value of 1.5. This value determines the clustering stringency, and is supposed to be a good balance between sensitivity and selectivity (Li et al., 2003). Then, protein BLAST were performed using NCBI BLAST+ version 2.2.29 (Altschul et al., 1997). Single copy orthologs were aligned using MAFFT v7.123b ; parameters: --auto --maxiterate 100). A phylogenetic tree was reconstructed with concatenated alignments using FastTree version 2.1.7 (Price, Dehal, & Arkin, 2010; parameters: -gamma -spr 4 -mlacc 2 -slownni). Protein sequence identity values were calculated based on pairwise Needleman-Wunsch global alignments made with needle (EMBOSS package version 6.5.7.0, Rice et al.). Gaps were not considered in the calculations.

**β-galactosidase assays**

β-galactosidase assays were adapted from Miller (1972) and performed at 28°C. Top10 *E. coli* strains were cultivated at 37°C. At OD_600nm_ between 0.5-0.8, IPTG 1mM or arabinose 0.02% were added during 3 hours to induce the production of Euo (from the pSRK-Gm vector)(Khan et al 2008), PhoB (from the pBAD22 vector) (Guzman et al 1995), and HrcA (from pBAD22). Fifty μL of bacteria were mixed with 750 μL of Z buffer and then lysed by addition of 10 μL of SDS and 20 μL of chloroform. 200 μL of ONPG (o-Nitrophenyl β-D-galactopyranoside; Sigma Aldrich, St-Louis, MO) were added to each reaction mixture and the time was recorded. When a yellow colour appeared, the reactions were stopped by addition of 500 μL of Na_2_CO_3_ and the times were recorded. The OD_600nm_ and OD_420nm_ of the supernatants were determined and the Millers units calculated using the following formula: U= (OD_420nm_ × 1,000)/(OD_600nm_ × time (in minutes) × volume of culture (in mL)). All tests were done in triplicate. The averages and the standard deviations were calculated and normalized according to the empty plasmid control (100%).

**Electrophoretic mobility shift assays (EMSAs)**

His_6_-Euo and His_6_-HrcA were produced from a BL21 (DE3) pLysS *E. coli* transformed with pET28a-Euo or HrcA. A culture at an OD600 between 0.5-0.8 was induced with 1mM of isopropyl-β-D-thiogalactoside (IPTG; Axonlab, Le Mont-sur-Lausanne, Switzerland) during 3 hours at 37°C. Cells were harvested by centrifugation at 3000g during 10 minutes and then lysed in FastBreak cell lysis solution (Promega, Madison, WI). The lysed cells were centrifuged 5 minutes at maximal speed and the supernatant were collected. His-tagged proteins were purified, in native conditions, using the MagneHis protein purification system (Promega, Madison, WI) according to the manufacturer’s instruction. Protein concentrations were determined by the quick start Bradford (BioRad, Reinach, Switzerland). His_6_-HrcA was always freshly prepared before proceeding to EMSA analysis.

For EMSA, 80 ng of DNA fragments (Supplementary Table 6-7) were incubated during 30 minutes at RT with an increasing concentration of purified protein in the reaction buffer (50mM Tris pH 7.5, 100 mM KCl, 10% Glycerol, BSA 1mg/mL, 1mM β-mercaptoethanol) in a final volume of 20 μL. All reactions were done in presence of non-specific competitor DNA, 1800 ng of Calf Thymus DNA (Invitrogen, Zug, Switzerland) for Euo and 800 ng of polydIdC (Sigma Aldrich, Buchs, Switzerland) for HrcA. The TBE 5% polyacrylamide gels (BioRad, Hercules, CA) were pre-run at 100V during 45 minutes. The samples were then separated at 100V during 1 hour. The DNA was detected after 5 minutes incubation in TBE with GelRed diluted 1/10000 using GeneGenius Imaging System (Syngene, Cambridge, UK) or for 5’Cy5-labelled PCR fragments using Typhoon Trio Imager (GE Healthcare). For EMSA detected using GelRed, the colours were inverted using ImageJ.

**References:**

Bailey TL, Elkan C (1995). The value of prior knowledge in discovering motifs with MEME. *Proceedings / International Conference on Intelligent Systems for Molecular Biology ; ISMB International Conference on Intelligent Systems for Molecular Biology* **3:** 21-29.

Bailey TL, Williams N, Misleh C, Li WW (2006). MEME: discovering and analyzing DNA and protein sequence motifs. *Nucleic Acids Res* **34:** W369-373.

Croxatto A, Murset V, Chassot B, Greub G (2013). Early expression of the type III secretion system of Parachlamydia acanthamoebae during a replicative cycle within its natural host cell Acanthamoeba castellanii. *Pathogens and disease* **69:** 159-175.

Fumeaux C, Radhakrishnan SK, Ardissone S, Theraulaz L, Frandi A, Martins D *et al* (2014). Cell cycle transition from S-phase to G1 in Caulobacter is mediated by ancestral virulence regulators. *Nature communications* **5:** 4081.

Greub G, Mege JL, Raoult D (2003). Parachlamydia acanthamoebae enters and multiplies within human macrophages and induces their apoptosis [corrected]. *Infect Immun* **71:** 5979-5985.

Guzman LM, Belin D, Carson MJ, Beckwith J (1995). Tight regulation, modulation, and high-level expression by vectors containing the arabinose PBAD promoter. *J Bacteriol* **177:** 4121-4130.

Khan SR, Gaines J, Roop RM, 2nd, Farrand SK (2008). Broad-host-range expression vectors with tightly regulated promoters and their use to examine the influence of TraR and TraM expression on Ti plasmid quorum sensing. *Appl Environ Microbiol* **74:** 5053-5062.

Radhakrishnan SK, Pritchard S, Viollier PH (2010). Coupling prokaryotic cell fate and division control with a bifunctional and oscillating oxidoreductase homolog. *Dev Cell* **18:** 90-101.

Thanbichler M, Iniesta AA, Shapiro L (2007). A comprehensive set of plasmids for vanillate- and xylose-inducible gene expression in Caulobacter crescentus. *Nucleic Acids Research* **35:** e137.
